# A phase I/II study of arfolitixorin and 5-fluorouracil in combination with oxaliplatin (plus or minus bevacizumab) or irinotecan in metastatic colorectal cancer

**DOI:** 10.1016/j.esmoop.2022.100589

**Published:** 2022-09-29

**Authors:** G. Carlsson, A. Koumarianou, T.K. Guren, J. Haux, P. Katsaounis, N. Kentepozidis, P. Pfeiffer, M. Brændengen, D. Mavroudis, H. Taflin, L. Skintemo, R. Tell, C. Papadimitriou

**Affiliations:** 1 Department of Surgery, University of Gothenburg, Sahlgrenska University Hospital/Östra Institute of Clinical Sciences, Gothenburg, Sweden; 2 Hematology Oncology Unit, Fourth Department of Internal Medicine, Attikon University Hospital, National and Kapodistrian University of Athens, Medical School, Athens, Greece; 3 Department of Oncology, Oslo University Hospital, Oslo, Norway; 4 Department of Surgery, Skaraborg Hospital, Skövde, Sweden; 5 Oncology Department, Metropolitan General Hospital, Athens, Greece; 6 Department of Medical Oncology, 251 General Air Force Hospital, Athens, Greece; 7 Experimental Research in Medical Cancer Therapy, Odense University Hospital, Odense, Denmark; 8 Department of Medical Oncology, University Hospital of Heraklion, Heraklion, Crete, Greece; 9 Department of Transplant Surgery, The Institute of Clinical Sciences, The Sahlgrenska Academy At University of Gothenburg, Gothenburg, Sweden; 10 Isofol Medical AB, Gothenburg, Sweden; 11 Oncology Unit, ‘Aretaieion’ University Hospital, National and Kapodistrian University of Athens, Athens, Greece

**Keywords:** arfolitixorin, chemotherapy, colorectal, fluorouracil, folate, [6R]-5,10-methylentetrahydrofolate

## Abstract

**Background:**

5-fluorouracil (5-FU) combined with a folate remains an essential treatment component for metastatic colorectal cancer (mCRC). Leucovorin is the folate most often used, but requires intracellular conversion to a reduced folate, and has high pharmacokinetic variability and limited bioavailability in patients with low folate pathway gene expression. Arfolitixorin is an immediately active form of folate, [6R]-5,10-methylenetetrahydrofolate ([6R]-MTHF), and may improve outcomes.

**Patients and methods:**

This open-label, multicenter, phase I/II study in patients with mCRC (NCT02244632) assessed the tolerability and efficacy of first- or second-line arfolitixorin (30, 60, 120, or 240 mg/m^2^ intravenous) with 5-FU alone, or in combination with oxaliplatin (plus or minus bevacizumab) or irinotecan, every 14 days. Safety, efficacy, and pharmacokinetics were assessed before and after four cycles (8 weeks) of treatment.

**Results:**

In 105 treated patients, investigators reported 583 adverse events (AEs) in 86 patients (81.9%), and 256 AEs (43.9%) were potentially related to arfolitixorin and 5-FU. Dose adjustments were required in 16 patients (15.2%). At 8 weeks, 9 out of 57 patients assessed for efficacy achieved an objective response (15.8%), and all 9 achieved a partial response. Six of these nine patients had received arfolitixorin as a first-line treatment. A further 33 patients (57.9%) achieved stable disease. Pharmacokinetics were assessed in 35 patients. The average t_max_ was 10 min, and area under the plasma concentration–time curve from time 0 to 1 h increased linearly between 30 and 240 mg/m^2^. No accumulation was observed for [6R]-MTHF following repeated administration, and there were no major pharmacokinetic differences between cycle 1 and cycle 4 at any dose.

**Conclusions:**

Arfolitixorin is a well-tolerated moderator of 5-FU activity. It is suitable for further investigation in mCRC and has the potential to improve treatment outcomes in patients with low folate pathway gene expression. Arfolitixorin can easily be incorporated into current standard of care, requiring minimal changes to chemotherapy regimens.

## Highlights

• Arfolitixorin is an immediately active form of folate and may improve outcomes with 5-FU-based chemotherapy.
• This phase I/II clinical trial demonstrated that arfolitixorin is a well-tolerated and effective folate agent in mCRC.
• Arfolitixorin can easily be incorporated into the current standard of care and is suitable for further investigation.

## Introduction

Colorectal cancer (CRC) is the third most common cancer, with a global incidence of 1.85 million new cases every year.^1^ The 5-year relative survival rate is 65%^2^ and ranges from 90% for patients with localized disease to 14% for those with metastatic CRC (mCRC).^3^ However, only 37% of CRC patients are diagnosed with local disease.^4^ Approximately half of all patients with CRC develop metastases,^5^ highlighting the need for an effective treatment strategy.

First-line treatment for localized unresectable mCRC commonly includes 5-fluorouracil (5-FU) combined with a folate agent.^6^^,^^7^ This combination improves response rates significantly compared with single-agent 5-FU.^8^^,^^9^ More recent phase III trials have investigated whether the clinical response can be further improved by adding other agents, including monoclonal antibodies, to the FOLFOX or FOLFIRI chemotherapy regimens. The TRIBE and TRIBE 2 studies demonstrated the benefit of FOLFOXIRI plus bevacizumab as a combination treatment in mCRC.^10^^,^^11^ The CALGB/SWOG 80405 trial found that overall survival was comparable with the addition of either cetuximab or bevacizumab to mFOLFOX6 or FOLFIRI chemotherapy.^12^ In the VOLFI study, response rates were improved by adding panitumumab to mFOLFOXIRI in *RAS* wild-type mCRC.^13^ There is rationale for evaluating the ways of optimizing outcomes with combination treatments.

5-FU suppresses DNA synthesis and triggers apoptosis by forming a cytotoxic ternary complex between its metabolite, 5-flurodeoxyuridine monophosphate (FdUMP), and thymidylate synthase (TS). Folinic acid [a precursor to the active substance 5,10-methylenetetrahydrofolate (MTHF)] substantially increases the affinity of FdUMP for TS, prolonging its inhibition by stabilizing the ternary complex.^14^, ^15^, ^16^ MTHF strengthens and prolongs the effects of 5-FU, enhancing cytotoxicity.^14^^,^^17^ Interest in the use of folate-based 5-FU chemotherapy in mCRC increased in the 1990s, driven by a landmark study which demonstrated that the addition of folate significantly improved response and progression rates in mCRC.^18^ Leucovorin is the most commonly used folate agent in mCRC, but requires metabolic activation. Variation in folate pathway gene expression leads to inconsistencies in the concentration of leucovorin reached in tumor tissue.^19^ Low or intermediate expression is linked to poor conversion and lower progression-free survival (PFS).^19^, ^20^, ^21^ Rationale exists for further exploration of a novel folate agent to improve the clinical effectiveness of 5-FU-based chemotherapy.

**Figure 1:**
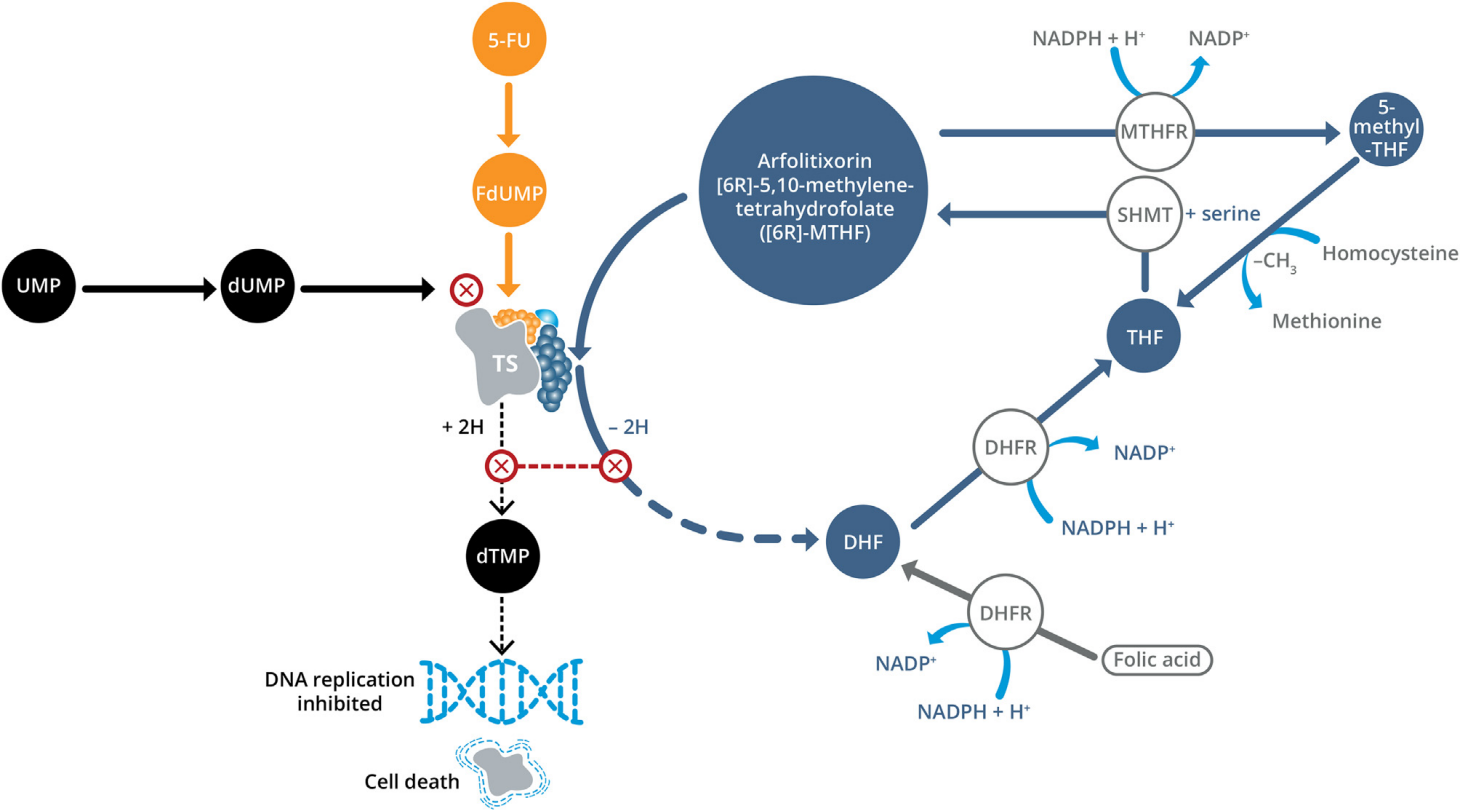
**Mechanism of action of arfolitixorin (stabilization of the ternary complex).** 5-FU, 5-fluorouracil; DHF, dihydrofolate; DHFR, dihydrofolate reductase; dTMP, deoxythymidine monophosphate; dUMP, deoxyuridine monophosphate; FdUMP, flurodeoxyuridine monophosphate; MTHFR, methylenetetrahydrofolate; NADPH, nicotinamide adenine dinucleotide phosphate; SHMT, serine hydroxymethyltransferase; THF, tetrahydrofolate; TS, thymidylate synthase; UMP, uridine monophosphate.

Arfolitixorin is a stable, immediately active form of [6R]-MTHF, which is a co-factor in the formation of the ternary complex (Figure 1).^17^^,^^22^ Its bioavailability is not influenced by folate gene expression, potentially making it a more effective folate agent.^23^ Arfolitixorin is metabolized to methyl-tetrahydrofolate (methyl-THF), which donates a methyl group in the remethylation of homocysteine to methionine,^24^ and THF, an active form of folic acid.^25^ Earlier studies of arfolitixorin^26^^,^^27^ have indicated that arfolitixorin has a promising pharmacological profile, and have laid the foundation for its more comprehensive evaluation in mCRC. This phase I/IIa multicenter study investigated clinical and pharmacokinetic (PK) outcomes for escalating doses of arfolitixorin combined with 5-FU, either alone or in combination with other agents, and aimed to determine a suitable dose for further investigation.

## Patients and methods

### Study design

This open-label, non-randomized, multicenter, phase I/IIa tolerability study (ClinicalTrials.gov identifier: NCT02244632) was conducted between September 2014 and January 2020 at 10 clinical centers across Europe: one in Denmark, five in Greece, two in Norway, and two in Sweden. The study was conducted in accordance with the Declaration of Helsinki (2008), as well as Good Clinical Practice, and applicable regulatory and local guidelines. The research ethics committees at each site approved the study and all patients provided written informed consent.

The study enrolled patients aged ≥18 years with stage IV mCRC verified by biopsy taken from either the primary tumor or a metastatic site. Eligible patients were required to have an Eastern Cooperative Oncology Group performance status (ECOG PS) of 0-2; a life expectancy exceeding 3 months; measurable disease on the RECIST version 1.1; and adequate hematologic, renal, and hepatic function. Detailed inclusion and exclusion criteria are available in Supplementary Material, available at https://doi.org/10.1016/j.esmoop.2022.100589.

The primary objective was to characterize the tolerability of four doses of arfolitixorin, in combination with 5-FU-based chemotherapy, in first-, second-, or later-line treatment by evaluating toxicity over 8 weeks of treatment. Secondary objectives were to establish the adverse event (AE) profile of arfolitixorin, abnormal laboratory results of clinical significance, tumor response after 8 weeks of therapy and disease progression, and the PK characteristics of the active substance [6R]-MTHF and metabolites methyl-THF and THF, in plasma following arfolitixorin administration on day 1 in cycles 1 and 4.

### Treatments

**Figure 2:**
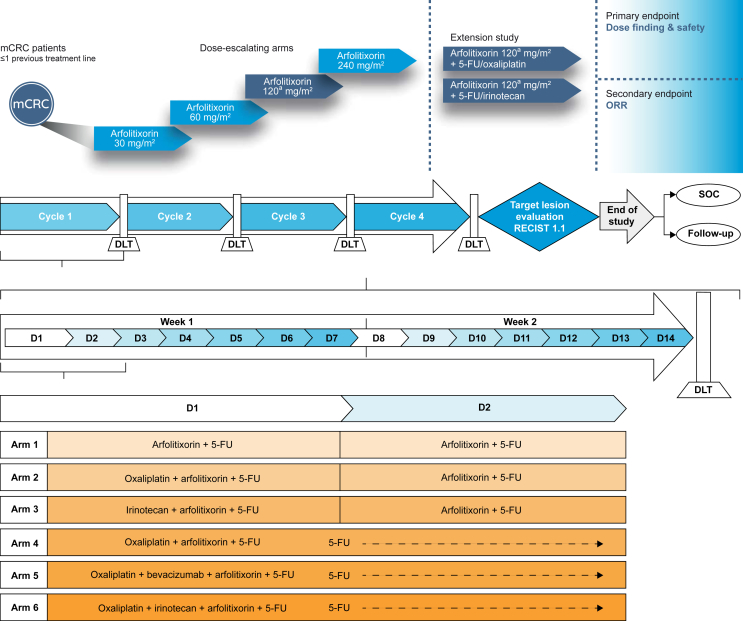
**Studydesign and dosing schedule.**^a^The dose of arfolitixorin 120 mg/m^2^ (given as two intravenous bolus injections 30 min apart) was selected as the dose for further investigation. 5-FU, 5-fluorouracil; DLT, dose-limiting toxicity; mCRC, metastatic colorectal cancer; SOC, standard of care.

Enrolled patients were assigned in cohorts to one of six investigational treatment arms (Figure 2). Each arm comprised treatment with arfolitixorin plus 5-FU alone or in combination with either oxaliplatin (with or without bevacizumab depending on the arm), or irinotecan. With exception to cohorts 18 and 19, each cohort aimed to enroll at least three patients, and the study design permitted expanding each cohort to enroll up to six patients (or five in cohorts 18 and 19). Chemotherapy treatments were given in four cycles, each lasting 2 weeks. Patients in each arm received 5-FU [either a 500-mg/m^2^ intravenous (i.v.) bolus injection on days 1 and 2, or a 400-mg i.v. bolus injection on day 1 plus a continuous 2400-mg/m^2^ infusion over 46 h] in combination with arfolitixorin (30, 60, 120, or 240 mg/m^2^ as either an i.v. bolus injection on days 1 and day 2, or 60, 120, or 240 mg/m^2^ as two i.v. bolus injections on day 1 only) with a stepwise dose-escalation strategy. Patients in arms 2, 4, and 5 also received oxaliplatin 85 mg/m^2^ for 15-20 min on day 1 (plus bevacizumab 5 mg/kg in arm 5), and patients in arms 3 and 6 received irinotecan 180 mg/m^2^ on day 1 (Figure 2).

Treatment arm 4 was based on the ARFOX regimen (oxaliplatin 85 mg/m^2^ on day 1; 5-FU as a 400-mg/m^2^ i.v. bolus injection on day 1 plus a continuous 2400-mg/m^2^ infusion over 46 h; and arfolitixorin 60, 120, or 240 mg/m^2^ on day 1 as two i.v. bolus injections), arm 5 was based on a modified ARFOX regimen (ARFOX plus bevacizumab), and arm 6 was based on the ARFIRI regimen [irinotecan 180 mg/m^2^ on day 1, 5-FU as a 400 mg/m^2^ i.v. bolus injection on day 1 followed by a continuous 2400-mg/m^2^ infusion over 46 h, and arfolitixorin 120 mg/m^2^ (the dose selected for further exploration) on day 1 as two i.v. bolus injections]. Dose adjustments were permitted for 5-FU, oxaliplatin, and irinotecan, but not for bevacizumab. De-escalation of arfolitixorin was permitted for the first three patients enrolled in cohort 1.

The selected dose for further exploration was determined by the efficacy and initial safety results from each cohort (62 patients in total who were originally enrolled). This dose was then evaluated in 43 additional patients, consisting of 22 patients in arm 4 and a further 21 patients in arm 6, to provide further data specifically on safety outcomes with the chosen dose.

### Safety, efficacy, and pharmacokinetic assessments

The safety profile of arfolitixorin in the chemotherapeutic combinations of interest was established based on the incidence and severity of AEs, and treatment-related dose-limiting toxicities (DLTs), from the start of treatment administration to the completion of study participation. AEs were assessed using the National Cancer Institute Common Terminology Criteria for Adverse Events (version 4.0). AEs of special interest (AESIs) were defined as AEs that differed from those anticipated for the therapies used and that, due to their severity or frequency, affected the current treatment schedule or led to delays or premature treatment discontinuation. An AE was classified as a DLT when it required cessation, or dose reduction, of one or more chemotherapeutic agents used in this study at any time during the study period. Biochemical parameters outside the normal range were considered AEs if they were deemed to be clinically significant. Evaluating the dose adjustments needed for each agent helped establish the tolerability of each tested regimen.

Efficacy was evaluated after 8 weeks of treatment based on the change in the size of the primary tumor after four treatment cycles, as well as evidence of disease progression (e.g. new metastatic growth). This was determined using computed tomography (CT) or magnetic resonance imaging scans of the thorax, abdomen, and pelvis, evaluated according to RECIST version 1.1. Specific efficacy endpoints included the objective response rate (ORR) and the number of partial responses (PRs) and complete responses. Early tumor shrinkage (ETS) was defined as a reduction of at least 20% in tumor size at week 8.

PK analyses for [6R]-MTHF (the active constituent of arfolitixorin) as well as metabolites THF and methyl-THF were carried out for a subset of patients at three centers with suitable facilities. Blood samples were taken directly before arfolitixorin administration, and at 10 min, and 1, 2, and 4 h after administration on day 1 of cycles 1 and 4. For treatment arms 4 and 5, because arfolitixorin was given as two i.v. bolus injections with a 30-min interval, PK blood samples were taken at 10 min, and 1, 2, and 4 h after the second dose (40 min, and 1.5, 2.5, and 4.5 h after the first dose, respectively).

PK blood samples were processed at a specialized laboratory in the UK. In accordance with the analytical protocol, plasma was immediately isolated from the blood by centrifugation at 1520 *g* (4°C, 10 min) and kept frozen at –80°C. Analyses were conducted using a validated liquid chromatography and mass spectrometry method. The PK endpoints included plasma concentrations at 10 min after arfolitixorin administration (C_10min_), the area under the plasma concentration–time curve from time 0 to time *t* [AUC_(0−t)_], calculated using the log-linear trapezoidal rule, and the dose-corrected AUC for the interval 0–*t* [AUC_(0−t)_/dose]. The lower limit of quantitation was set at 250 μg/l for THF and 100 μg/l for [6R]-MTHF and 5-methyl-THF. Data of concentration against time were evaluated by a non-compartmental analysis, which incorporated the actual timepoints when blood samples were taken and the dose levels. All PK analyses were carried out using Phoenix® WinNonlin® version 8.2, build 8.2.0.4383 (Certara, Princeton, NJ).

### Statistical analysis

Descriptive statistics were carried out to analyze continuous variables, including calculating the mean and the range. Categorical data were summarized as counts and percentages. Formal statistical tests of comparison were not carried out. The data analysis set for safety endpoints consisted of all enrolled patients who received at least one dose of arfolitixorin. The data analysis set for efficacy endpoints consisted of patients who received at least one dose of arfolitixorin and had at least one post-baseline assessment of any efficacy variable (the full analysis dataset). For PK parameters, geometric means and coefficients of variation were calculated. All statistical analyses were conducted using SAS® version 9.4 (Cary, NC).

## Results

### Patient characteristics

Overall, 105 patients were enrolled in this study. This comprised 62 in the original dose-finding cohort and 43 who were subsequently recruited, including an additional 11 patients in arm 5 (ARFOX plus bevacizumab) when arfolitixorin 120 mg/m^2^ (two i.v. bolus doses of 60 mg/m^2^) was selected as the dose for further investigation. The median age was 66 years, and almost all patients were of Caucasian ethnicity (98.1%). Over three-quarters of patients (78.1%) had an ECOG PS of 0, and the remainder had an ECOG PS of 1 or 2. Most patients received arfolitixorin as either a first- or second-line treatment (57.1% and 33.3%, respectively) (Table 1). Information on treatment allocation is included in Supplementary Table S1, available at https://doi.org/10.1016/j.esmoop.2022.100589.

**Table 1:** Summary of patient demographics

	Arfolitixorin dose (mg/m^2^)				Overall
	30	60	120	240	
Total (*n*)	12	21	65	7	105
Female, *n* (%)	4 (33.3)	11 (52.4)	32 (49.2)	4 (57.1)	51 (48.6)
Male, *n* (%)	8 (66.7)	10 (47.6)	33 (50.8)	3 (42.9)	54 (51.4)
Race					
White, *n* (%)	12 (100)	21 (100)	64 (98.5)	6 (85.7)	103 (98.1)
Asian, *n* (%)	—	—	1 (1.5)	1 (14.3)	2 (1.9)
Characteristics					
Age, mean years (range)	66 (37-84)	62 (47-77)	63 (32-85)	68 (45-85)	64 (32-85)
Height, mean cm (range)	177 (162-195)	171 (155-189)	169 (155-187)	169 (160-182)	171 (155-195)
Weight, mean kg (range)	82 (61-106)	76 (45-100)	75 (45-115)	72 (60-95)	76 (45-115)
ECOG PS					
0, *n* (%)	10 (83.3)	16 (76.2)	52 (80.0)	4 (57.1)	82 (78.1)
1, *n* (%)	2 (16.7)	4 (19.1)	13 (20.0)	3 (42.9)	22 (21.0)
2, *n* (%)	—	1 (4.8)	—	—	1 (1.0)
Line of treatment					
First, *n* (%)	6 (50.0)	7 (33.3)	45 (69.2)	2 (28.6)	60 (57.1)
Second, *n* (%)	4 (33.3)	9 (42.9)	17 (26.2)	5 (71.4)	35 (33.3)
Third, *n* (%)	2 (16.7)	5 (23.8)	3 (4.6)	—	10 (9.5)

ECOG PS, Eastern Cooperative Oncology Group performance status.

### Safety and tolerability outcomes

During the 8-week treatment course, investigators reported 583 AEs in 86 (81.9%) of the 105 patients in the safety analysis set. The most common AEs of all grades were nausea/vomiting (88 events), diarrhea (40 events), and fatigue (40 events). Overall, 74 AEs (12.7%) were grade ≥3; the most common were neutropenia (20 events) and pain (10 events). A total of 48 AEs (8.2%) were categorized as DLTs.

Of the 583 AEs, 256 (43.9%) were potentially related to arfolitixorin and 5-FU, 67 (11.5%) to 5-FU but not arfolitixorin, and 1 AE (0.17%) to arfolitixorin but not 5-FU, which was a case of grade 1 skin lesions on one hand. Patients receiving oxaliplatin (*n* = 63) experienced 337 AEs and patients receiving irinotecan (*n* = 29) experienced 171 AEs. Of these, 225 (66.8%) and 107 (62.6%) were potentially related to each chemotherapy agent, respectively. Nine AEs in six patients were potentially related to bevacizumab, 5-FU, and oxaliplatin. Only one AE, which was a case of proteinuria, was considered related to bevacizumab alone. The remaining eight AEs were most commonly hematologic and all of grade 1 or 2 severity.

Investigators reported 82 AESIs in 36 patients (34.3%) overall (Table 2), with 62 AESIs potentially related to arfolitixorin or 5-FU (75.6%), and none potentially related to arfolitixorin only. Of these, 37 (59.7%) were grade ≥3 in severity and were predominantly hematologic. One patient in arm 2 (5-FU plus oxaliplatin) treated with 60 mg/m^2^ arfolitixorin terminated treatment prematurely due to a DLT of grade 2 neutropenia. This patient also experienced grade 2 diarrhea and grade 3 fatigue, both of which were AESIs but not DLTs. Another patient in this arm who received 30 mg/m^2^ arfolitixorin terminated treatment owing to an AE of grade 2 malaise, which was not classified as an AESI or a DLT. As a result, three additional patients were recruited into arm 2 during the study. Two patients in arm 4 (120 mg/m^2^ arfolitixorin) experienced non-dose-limiting AESIs of increased serum creatinine (all of grade ≤3), one of whom also experienced acute renal failure (grade 3). These AESIs led to the addition of three patients to arm 4. No cases of creatinine increase were reported in any other study participants.

**Table 2:** The number of AESIs by arfolitixorin dose and AE grade according to CTCAE Version 4.0

	Arfolitixorin dose mg/m^2^								
	30		60		120		240		Total
AESI	Grade 1/2	Grade ≥3	Grade 1/2	Grade ≥3	Grade 1/2	Grade ≥3	Grade 1/2	Grade ≥3	
Neutropenia	—	5	5	6	7	7	—	—	30
Leukopenia	—	—	—	1	5	—	—	—	6
Increased creatinine	—	—	—	—	4	2	—	—	6
Nausea	—	—	—	—	3	2	—	—	5
Vomiting	—	—	—	—	3	2	—	—	5
Fatigue	1	—	—	1	1	1	—	1	5
Thrombocytopenia	—	—	—	—	3	1	—	—	4
Anemia	—	—	1	—	2	—	—	—	3
Diarrhea	—	—	1	—	1	1	—	—	3
Febrile neutropenia	—	—	—	1	—	1	—	—	2
Dehydration	—	—	—	—	—	2	—	—	2
Hyponatremia	—	—	—	—	—	2	—	—	2
Ileitis	—	—	—	—	—	1	—	—	1
Infection	—	—	—	—	—	—	—	1	1
Mesenteric vein thrombosis	—	1	—	—	—	—	—	—	1
Acute renal failure	—	—	—	—	—	1	—	—	1
Low platelet count	—	—	—	—	1	—	—	—	1
Abdominal pain	—	—	—	—	—	1	—	—	1
Pancolitis	—	—	—	—	—	1	—	—	1
Syncope	—	—	—	—	—	1	—	—	1
Progression of pre-existing cancer	—	—	—	—	—	1	—	—	1
									82

AE, adverse event; AESI, adverse event of special interest; CTCAE, Common Terminology Criteria for Adverse Events.

Dose adjustments for any administered chemotherapy agent were required in 16 out of 105 patients (15.2%). This included 14 (22.6%) of 62 originally enrolled patients, and 2 (4.7%) of 43 subsequently recruited patients. Most of these patients only required one dose adjustment (*n* = 12, 11.4%); however, two dose adjustments were required in three patients (2.9%) and three dose adjustments were required in one patient (1.0%). The latter patient received 85 mg/m^2^ oxaliplatin, 5-FU as a 400 mg/m^2^ i.v. bolus injection on day 1 followed by a continuous 2400-mg/m^2^ infusion over 46 h, and two i.v. bolus injections of arfolitixorin 120 mg/m^2^.

Four deaths occurred after the first dose of the study drug, two of which were due to disease progression in patients in arm 1 (5-FU plus arfolitixorin) and arm 6 (ARFIRI). Both events were considered related to the patients’ underlying disease. For the remaining two deaths, one was due to a pulmonary embolism with acute respiratory distress syndrome in a patient in arm 6. This was related to progressive pulmonary disease which was not considered treatment related, and the other was due to an intestinal perforation in a patient in arm 5 (ARFOX plus bevacizumab) during the follow-up study, which was considered related to bevacizumab.

### Efficacy outcomes

**Figure 3:**
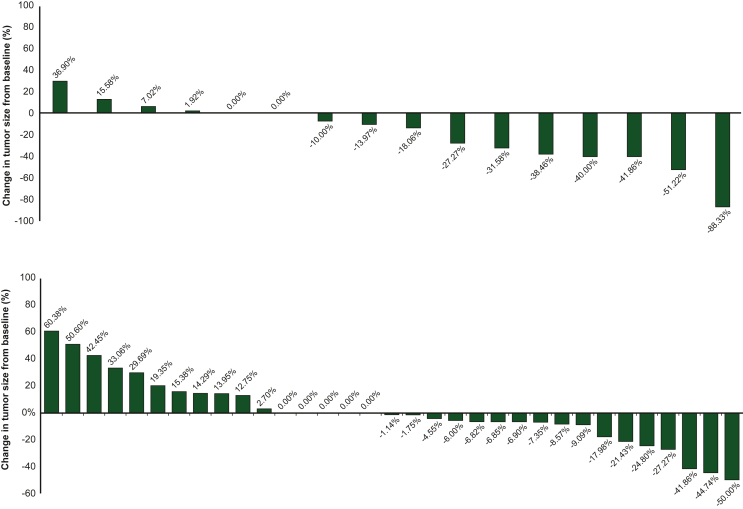
**Percentage change in tumor size at week 8 in the 16 patients who received arfolitixorin as first-line treatment (top), and in the 33 patients who received arfolitixorin as second-line treatment (bottom).** (57 of 62 patients were assessable for tumor response with both pre- and post-baseline target lesion measurements. Each bar represents one patient).

Efficacy outcomes were evaluated in 57 out of the 62 patients enrolled in the original dose-finding study (before the subsequent enrollment of additional patients). The remaining five patients did not complete treatment and were excluded from analyses. At 8 weeks, an objective response (OR) was achieved in nine patients (15.8%), all of whom were graded as PR. A higher proportion of patients who achieved an OR received arfolitixorin as a first-line treatment (6 out of the 17 patients treated first-line, 35.3%), compared with second-line treatment (3 out of the 33 patients treated second-line, 9.1%). Arfolitixorin 60 mg/m^2^ was the dose that yielded the most ORs. A PR was achieved in one patient (1.8% of total evaluated), six patients (10.5%), one patient (1.8%), and one patient (1.8%) who received arfolitixorin 30, 60, 120, and 240 mg/m^2^, respectively. A further 33 patients (57.9%) achieved stable disease (SD) at 8 weeks. Tumor responses analyzed on CT scans indicated that 14 patients (24.6%) exhibited ETS (Figure 3).

### PK outcomes

This study investigated the PK of arfolitixorin as a secondary objective in a subset of 53 patients. The t_max_ of the active metabolite [6R]-MTHF was reached rapidly [at 10 min in arms 1-3 or 40 min (i.e. 10 min after the second dose) in arms 4-6] (Supplementary Figure S1, available at https://doi.org/10.1016/j.esmoop.2022.100589), suggesting a rapid onset of action. However, since no blood samples were available between 0 and ∼40 min in arms 4-6, the AUC could not be reliably determined. The last blood sample with detectable levels of [6R]-MTHF was at ∼1 h after dosing, and there was no indication that [6R]-MTHF accumulated between doses (Supplementary Table S2, available at https://doi.org/10.1016/j.esmoop.2022.100589). The PK findings were all within the expected range. Full details of PK outcomes, including the metabolites THF (Supplementary Figure S2, available at https://doi.org/10.1016/j.esmoop.2022.100589 and Supplementary Table S3, available at https://doi.org/10.1016/j.esmoop.2022.100589) and methyl-THF (Supplementary Figure S3, available at https://doi.org/10.1016/j.esmoop.2022.100589 and Supplementary Table S4, available at https://doi.org/10.1016/j.esmoop.2022.100589), are included in the Supplementary Material, available at https://doi.org/10.1016/j.esmoop.2022.100589.

## Discussion

This multicenter, phase I/IIa study evaluated the safety, tolerability, and efficacy of arfolitixorin combined with 5-FU alone or with oxaliplatin (plus or minus bevacizumab) or irinotecan in mCRC for 8 weeks. The study met its primary objective of characterizing the tolerability of arfolitixorin in stage IV mCRC over this 8-week period, as well as its secondary and PK endpoints. Safety and efficacy were assessed in 105 and 62 patients, respectively, who were enrolled in one of six treatment arms. The safety data indicate that arfolitixorin has a manageable safety and tolerability profile, and higher doses of arfolitixorin were not associated with a higher incidence of AEs; only one AE was reported as being related to arfolitixorin alone, which was an AE of grade 1 skin lesions. Overall, 42 (73.7%) out of 57 patients achieved disease control (either an OR or SD), indicating the high clinical effectiveness of arfolitixorin plus chemotherapy in mCRC. The proportion of patients who achieved an OR was numerically higher among those who received arfolitixorin as a first-line treatment compared with second-line (35.3% versus 9.1%), suggesting arfolitixorin may have a greater potentiating effect on 5-FU in the first-line setting.

The AE profiles of arms 1-3 (5-FU plus arfolitixorin alone or with oxaliplatin or irinotecan) were not noticeably different from the AE profiles in arms 4 (ARFOX), 5 (ARFOX plus bevacizumab), or 6 (ARFIRI). This indicates that arfolitixorin is well tolerated with different treatment combinations, although formal inter-group comparisons were not carried out. The most common any-grade AEs were nausea/vomiting, diarrhea, and fatigue, similar to the occurrence of AEs reported in other studies of chemotherapy agents in mCRC.^28^^,^^29^ The pattern of AEs in published studies of FU-based chemotherapy in mCRC suggests that their occurrence is associated more specifically with the chemotherapy agents that have been used, rather than the folate agent. In a 6-month randomized trial comparing 5-FU plus leucovorin with the FOLFOX regimen in CRC, AEs that are typically associated with chemotherapy, including neutropenia, diarrhea, nausea, and vomiting, were more common with FOLFOX than with 5-FU plus leucovorin.^30^ FOLFOX and FOLFIRI are standard regimens in mCRC and have well-defined safety profiles.^31^^,^^32^ The safety findings of this study support the published literature and indicate that arfolitixorin does not exacerbate the occurrence of AEs.

Leucovorin is an established folate agent in mCRC and has a well-known safety and tolerability profile.^33^ Some studies which evaluated the addition of irinotecan and oxaliplatin to the 5-FU/leucovorin regimen in the early 2000s demonstrated improved response and survival rates, but not all.^6^^,^^34^, ^35^, ^36^ Despite this, the clinical utility of leucovorin is limited as it requires bioconversion to 5-MTHF. Treatment response is poor in patients with low folate pathway gene expression, in whom low levels of 5-MTHF result in weak TS inhibition.^20^^,^^23^^,^^37^, ^38^, ^39^, ^40^ Substantial interindividual heterogeneity exists in the concentration of leucovorin in tumor tissues,^39^ and only about 20% of patients achieve a clinically meaningful response with 5-FU and leucovorin.^18^^,^^41^^,^^42^ There is a lack of evidence for an optimal dose of leucovorin and doses ranging from 20 mg/m^2^ to 500 mg/m^2^ i.v. have been evaluated in mCRC.^17^^,^^43^ High doses may be needed in patients with particular polymorphisms,^39^ but have been associated with a higher incidence of diarrhea.^33^ There is, therefore, considerable interest in developing an adjuvant folate agent that overcomes these limitations.

Arfolitixorin is an immediately active form of [6R]-MTHF. Tissue concentrations of [6R]-MTHF were relatively stable in this study, with evidence of steady formation and elimination. This complements earlier research observing stable therapeutic levels of [6R]-MTHF in tumor tissue and plasma from patients with mCRC after arfolitixorin dosing.^17^^,^^26^^,^^39^ Stable therapeutic levels of folate can reasonably be anticipated to maximize the treatment response. In this study, most patients achieved either PR (15.8%) or SD (57.9%). Furthermore, 25% of patients experienced ETS at week 8. ETS is a robust prognostic biomarker, and is associated with significant and clinically meaningful reductions in the risk of death and progression in mCRC.^44^ Overall, the efficacy results indicate that arfolitixorin is a well-tolerated and effective direct moderator of 5-FU activity, and is effective in the first-line setting.

The highest dose of arfolitixorin administered in this study was 240 mg/m^2^. There was no evidence of enhanced efficacy with this dose compared with lower doses, although only a small number of patients received this dose (*n* = 7), making it hard to draw conclusions. Arfolitixorin was well tolerated at each of the doses evaluated (30, 60, 120, and 240 mg/m^2^). Plasma concentrations of the biologically active molecule ([6R]-MTHF) and its metabolites (THF and methyl-THF) increased linearly with the dose increase of arfolitixorin from 30 to 240 mg/m^2^, indicating that higher plasma levels are reached with higher doses. On the basis of existing evidence associating folate plasma concentrations with early treatment response,^27^ a higher dose of arfolitixorin may elicit a greater treatment response than lower doses.

In the interest of balancing efficacy with tolerability, a dose of arfolitixorin 120 mg/m^2^ (given as two i.v. bolus injections 30 min apart) was selected as the dose for further investigation. This decision was also supported by a published subset analysis of 33 patients, who were initially enrolled into the PK analysis set of this trial.^27^ The analysis found that levels of plasma deoxyuridine, a surrogate marker for toxicity and early clinical response, were significantly higher 24 h into cycle 1 with arfolitixorin 120 mg/m^2^ than with 30 or 60 mg/m^2^.^27^ This suggests that arfolitixorin 120 mg/m^2^ elicits an earlier clinical response than with the lower doses of 30 and 60 mg/m^2^, and the findings of this study indicate that this dose has a manageable safety and tolerability profile.

Several factors may have influenced treatment outcomes. Combination treatment and the addition of other agents to the chemotherapy backbone, such as bevacizumab to FOLFOX/FOLFOXIRI, have known clinical benefits in mCRC.^10^^,^^11^ Without randomization, it is difficult to directly attribute the observed treatment effects to arfolitixorin. The method of administration is also relevant in this regard, and molecular analyses of CRC tumor tissues indicated that the method of 5-FU administration (bolus or continuous) induces different metabolic pathways with differential inhibitory effects.^45^^,^^46^ However, 5-FU has limited effectiveness without folate,^23^ and the results of this study indicate that arfolitixorin is suitable for use with a 5-FU treatment combination.

Other limitations of this phase I/IIa trial include the small sample sizes in each cohort, the short duration of follow-up, and the lack of formal statistical comparisons of outcomes between treatment arms. Future research should aim to elucidate the predictors of response to arfolitixorin, and whether factors such as age, stage of mCRC, or biomarker expression are suitable for this purpose. The phase III AGENT study (NCT03750786) is exploring the efficacy of arfolitixorin versus leucovorin in combination with 5-FU, oxaliplatin, and bevacizumab in patients with advanced mCRC. It will provide further efficacy data, including ORR (primary outcome), PFS, and duration of response.

Overall, this study demonstrates that arfolitixorin has a manageable safety and tolerability profile with reasonable treatment outcomes when combined with 5-FU-based chemotherapy in mCRC. Based on the results, arfolitixorin has the potential to improve outcomes in patients with low folate pathway gene expression, who may have a sub-optimal response to other folate agents, and is, therefore, suitable for further investigation.

## References

[1] Biller L.H., Schrag D. (2021). Diagnosis and treatment of metastatic colorectal cancer: a review. JAMA.

[2] Cancer.Net. Colorectal Cancer: Statistics. Published 2021. Available at https://www.cancer.net/cancer-types/colorectal-cancer/statistics. Accessed October 8, 2021.

[3] Siegel R.L., Miller K.D., Sauer A.G. (2020). Colorectal cancer statistics, 2020. CA Cancer J Clin.

[4] American Cancer Society. Colorectal Cancer Facts & Figures 2020-2022. Published 2020. Available at https://www.cancer.org/content/dam/cancer-org/research/cancer-facts-and-statistics/colorectal-cancer-facts-and-figures/colorectal-cancer-facts-and-figures-2020-2022.pdf. Accessed September 20, 2021.

[5] Väyrynen V., Wirta E.V., Seppälä T. (2020). Incidence and management of patients with colorectal cancer and synchronous and metachronous colorectal metastases: a population-based study. BJS Open.

[6] de Gramont A., Figer A., Seymour M. (2000). Leucovorin and fluorouracil with or without oxaliplatin as first-line treatment in advanced colorectal cancer. J Clin Oncol.

[7] Douillard J.Y., Cunningham D., Roth A.D. (2000). Irinotecan combined with fluorouracil compared with fluorouracil alone. as first-line treatment for metastatic colorectal cancer: a multicentre randomised trial. Lancet.

[8] Arbuck S.G. (1989). Overview of clinical trials using 5-fluorouracil and leucovorin for the treatment of colorectal cancer. Cancer.

[9] O’Connell M.J. (1989). A phase III trial of 5-fluorouracil and leucovorin in the treatment of advanced colorectal cancer. A Mayo Clinic/North Central Cancer Treatment Group study. Cancer.

[10] Cremolini C., Loupakis F., Antoniotti C. (2015). Early tumor shrinkage and depth of response predict long-term outcome in metastatic colorectal cancer patients treated with first-line chemotherapy plus bevacizumab: results from phase III TRIBE trial by the Gruppo Oncologico del Nord Ovest. Ann Oncol.

[11] Cremolini C., Antoniotti C., Rossini D. (2020). Upfront FOLFOXIRI plus bevacizumab and reintroduction after progression versus mFOLFOX6 plus bevacizumab followed by FOLFIRI plus bevacizumab in the treatment of patients with metastatic colorectal cancer (TRIBE2): a multicentre, open-label, phase 3, rand. Lancet Oncol.

[12] Venook A.P., Niedzwiecki D., Lenz H.J. (2017). Effect of first-line chemotherapy combined with cetuximab or bevacizumab on overall survival in patients with KRAS Wild-type advanced or metastatic colorectal cancer: a randomized clinical trial. JAMA.

[13] Modest D.P., Martens U.M., Riera-Knorrenschild J. (2019). FOLFOXIRI plus panitumumab as first-line treatment of RAS wild-type metastatic colorectal cancer: the randomized, open-label, phase II VOLFI study (AIO KRK0109). J Clin Oncol.

[14] Ullman B., Lee M., Martin D.W., Santi D.V. (1978). Cytotoxicity of 5 fluoro 2’ deoxyuridine: requirement for reduced folate cofactors and antagonism by methotrexate. Proc Natl Acad Sci U S A.

[15] Evans R.M., Laskin J.D., Hakala M.T. (1981). Effect of excess folates and deoxyinosine on the activity and site of action of 5-fluorouracil. Cancer Res.

[16] Machover D. (1997). A comprehensive review of 5-fluorouracil and leucovorin in patients with metastatic colorectal carcinoma. Cancer.

[17] Gustavsson B., Carlsson G., Machover D. (2015). A review of the evolution of systemic chemotherapy in the management of colorectal cancer. Clin Colorectal Cancer.

[18] Poon M.A., O’Connell M.J., Moertel C.G. (1989). Biochemical modulation of fluorouracil: evidence of significant improvement of survival and quality of life in patients with advanced colorectal carcinoma. J Clin Oncol.

[19] Odin E., Sondén A., Gustavsson B., Carlsson G., Wettergren Y. (2015). Expression of folate pathway genes in stage III colorectal cancer correlates with recurrence status following adjuvant bolus 5-FU-Based chemotherapy. Mol Med.

[20] Gustavsson B., Odin E., Saksena P. (2018). Folate gene prediction of treatment response to 5-FU and leucovorin in advanced colorectal cancer. J Clin Oncol.

[21] Ntavatzikos A., Spathis A., Patapis P. (2017). Integrating TYMS, KRAS and BRAF testing in patients with metastatic colorectal cancer. World J Gastroenterol.

[22] Parker W.B., Cheng Y.C. (1990). Metabolism and mechanism of action of 5-fluorouracil. Pharmacol Ther.

[23] Danenberg P.V., Gustavsson B., Johnston P. (2016). Folates as adjuvants to anticancer agents: chemical rationale and mechanism of action. Crit Rev Oncol Hematol.

[24] Hubner R.A., Houlston R.S. (2009). Folate and colorectal cancer prevention. Br J Cancer.

[25] Yoshii K., Hosomi K., Sawane K., Kunisawa J. (2019). Metabolism of dietary and microbial vitamin B family in the regulation of host immunity. Front Nutr.

[26] Wettergren Y., Taflin H., Odin E., Kodeda K., Derwinger K. (2015). A pharmacokinetic and pharmacodynamic investigation of Modufolin® compared to Isovorin® after single dose intravenous administration to patients with colon cancer: a randomized study. Cancer Chemother Pharmacol.

[27] Taflin H., Odin E., Carlsson G. (2020). Plasma deoxyuridine as a surrogate marker for toxicity and early clinical response in patients with metastatic colorectal cancer after 5-FU-based therapy in combination with arfolitixorin. Cancer Chemother Pharmacol.

[28] Glimelius B., Sørbye H., Balteskard L. (2008). A randomized phase III multicenter trial comparing irinotecan in combination with the Nordic bolus 5-FU and folinic acid schedule or the bolus/infused de Gramont schedule (Lv5FU2) in patients with metastatic colorectal cancer. Ann Oncol.

[29] Tveit K.M., Guren T., Glimelius B. (2012). Phase III trial of cetuximab with continuous or intermittent fluorouracil, leucovorin, and oxaliplatin (Nordic FLOX) versus FLOX alone in first-line treatment of metastatic colorectal cancer: the NORDIC-VII study. J Clin Oncol.

[30] André T., Boni C., Mounedji-Boudiaf L. (2004). Oxaliplatin, fluorouracil, and leucovorin as adjuvant treatment for colon cancer. N Engl J Med.

[31] Van Cutsem E., Cervantes A., Nordlinger B., Arnold D. (2014). The ESMO Guidelines Working Group. Metastatic colorectal cancer: ESMO clinical practice guidelines for diagnosis, treatment and follow-up. Ann Oncol.

[32] Benson A.B., Venook A.P., Al-Hawary M.M. (2018). NCCN Guidelines Insights: colon cancer, version 2.2018. J Natl Compr Cancer Netw.

[33] Hsu C.-Y., Chen C.-Y., Lin Y.-M., Tam K.-W. (2020). Efficacy and safety of high-dose vs low-dose leucovorin in patients with colorectal cancer: systematic review and meta-analysis. Color Dis.

[34] Saltz L.B., Cox J.V., Blanke C. (2000). Irinotecan plus fluorouracil and leucovorin for metastatic colorectal cancer. N Engl J Med.

[35] Rothenberg M.L., Oza A.M., Bigelow R.H. (2003). Superiority of oxaliplatin and fluorouracil-leucovorin compared with either therapy alone in patients with progressive colorectal cancer after irinotecan and fluorouracil-leucovorin: interim results of a phase III trial. J Clin Oncol.

[36] Giacchetti S., Perpoint B., Zidani R. (2000). Phase III multicenter randomized trial of oxaliplatin added to chronomodulated fluorouracil-leucovorin as first-line treatment of metastatic colorectal cancer. J Clin Oncol.

[37] Priest D.G., Schmitz J.C., Bunni M.A., Stuart R.K. (1991). Pharmacokinetics of leucovorin metabolites in human plasma as a function of dose administrated orally and intravenously. J Natl Cancer Inst.

[38] Odin E., Wettergren Y., Carlsson G., Gustavsson B. (2013). Determination of reduced folates in tumor and adjacent mucosa of colorectal cancer patients using LC-MS/MS. Biomed Chromatogr.

[39] Taflin H., Wettergren Y., Odin E., Carlsson G., Derwinger K. (2014). Folate levels and polymorphisms in the genes MTHFR, MTR, and TS in colorectal cancer. Clin Med Insights Oncol.

[40] Odin E., Sondén A., Carlsson G., Gustavsson B., Wettergren Y. (2019). Folate pathway genes linked to mitochondrial biogenesis and respiration are associated with outcome of patients with stage III colorectal cancer. Tumor Biol.

[41] Petrelli N., Douglass H.O., Herrera L. (1989). The modulation of fluorouracil with leucovorin in metastatic colorectal carcinoma: a prospective randomized phase III trial. Gastrointestinal Tumor Study Group. J Clin Oncol.

[42] Thirion P., Michiels S., Pignon J. (2004). Modulation of fluorouracil by leucovorin in patients with advanced colorectal cancer: an updated meta-analysis. J Clin Oncol.

[43] Van Cutsem E., Cervantes A., Adam R. (2016). ESMO consensus guidelines for the management of patients with metastatic colorectal cancer. Ann Oncol.

[44] Petrelli F., Pietrantonio F., Cremolini C. (2015). Early tumour shrinkage as a prognostic factor and surrogate end-point in colorectal cancer: a systematic review and pooled-analysis. Eur J Cancer.

[45] Matsusaka S., Yamasaki H., Kitayama Y., Okada T., Maeda S. (2003). Differential effects of two fluorouracil administration regimens for colorectal cancer. Oncol Rep.

[46] Kubota T., Watanabe M., Otani Y., Kitajima M., Fukushiuma M. (2002). Different pathways of 5-fluorouracil metabolism after continuous venous or bolus injection in patients with colon carcinoma: possible predictive value of thymidylate synthetase mRNA and ribonucleotide reductase for 5-fluorouracil sensitivity. Anticancer Res.

