# Supplementary material for: A phase I/II study of arfolitixorin and 5-fluorouracil in combination with oxaliplatin (plus or minus bevacizumab) or irinotecan in metastatic colorectal cancer
Source: ESMO Open. 2022 Sep 29;7(5):100589. doi: 10.1016/j.esmoop.2022.100589 (PMC9588906; doi:10.1016/j.esmoop.2022.100589)
Supplement: Supplementary Material [file mmc1.docx]

**Supplementary information**

Contents

[Eligibility Criteria 2](#_Toc109215610)

[Detailed inclusion criteria 2](#_Toc109215611)

[Detailed exclusion criteria 2](#_Toc109215612)

[Patient enrolment and treatment allocation 3](#_Toc109215613)

[**Table S1.** Patient enrollment and treatment allocation 3](#_Toc109215614)

[Pharmacokinetic Results 4](#_Toc109215615)

[[6R]-5,10-methylenetetrahydrofolate (active substance) 4](#_Toc109215616)

[**Figure S1.** Mean (± SD) plasma concentration versus time for arfolitixorin ([6R]-MTHF) (cycle 1 and cycle 4 combined data) divided by treatment arm according to arfolitixorin dose 5](#_Toc109215617)

[**Table S2**. Statistics of main pharmacokinetic (PK) parameters of parent compound MTHF (arfolitixorin) after I.V. bolus administration (Cycle 1 and Cycle 4 combined) 6](#_Toc109215618)

[THF (metabolite) 8](#_Toc109215619)

[**Figure S2**. Mean (± SD) plasma concentration vs time for tetrahydrofolate (THF) (Cycle 1 and Cycle 4 combined data) divided by treatment arm according to arfolitixorin dose 9](#_Toc109215620)

[**Table S3**. Statistics of main PK parameters of THF after IV bolus administration of arfolitixorin (Cycles 1 and 4 combined) 11](#_Toc109215621)

[Methyl-THF (metabolite) 13](#_Toc109215622)

[**Figure S3**. Mean (± SD) plasma concentration vs time for methyltetrahydrofolate (Methyl THF) (Cycle 1 and Cycle 4 combined data) divided by treatment arm according to arfolitixorin dose 15](#_Toc109215623)

[**Table S4**. Statistics of main PK parameters of Methyl-THF after IV bolus administration of arfolitixorin (Cycle 1 and Cycle 4 combined). 16](#_Toc109215624)

# Eligibility Criteria

## Detailed inclusion criteria

The patients had to meet all of the following criteria to be eligible to enter the study. Advanced metastatic colorectal (Stage IV) cancer verified by biopsy, eligible for first- or second-line therapy. Computerised tomography (CT) scan or MRI of thorax, abdomen and pelvis; within five (≤ 5) weeks before start of chemotherapy. Evaluable disease and one measurable site of disease according to RECIST 1.1 criteria (at least 10 mm for CT-scan or magnetic resonance imaging). Male and female adults, age of 18 years or older. WHO performance status 0 ≤ 2, and life expectancy ≥ 3 months. Adequate haematological function (haemoglobin ≥ 100 g/L, absolute neutrophil count ≥ 1.5 x 10^9^/L and platelets ≥100 x 10^9^/L), renal and hepatic function (creatinine clearance > 50 mL/min, total bilirubin ≤ 1.5 times upper limit of normal, aspartate transaminase and alanine transaminase ≤ 3 times upper limit of normal and ≤ 5 times upper limit of normal in case of liver metastases). Eligible for at least one of the chemotherapy treatment arms in the study protocol which, at the time of enrolment, is currently open for recruitment, according to Investigator’s judgement. Negative pregnancy test for females of child-bearing potential. Willing and able to provide informed consent. Using adequate contraceptive measures: Female patients who have been post-menopausal for more than one year or female patients of childbearing potential using a highly efficient method of contraception (i.e. a method with less than 1% failure rate [e.g. sterilization, hormone implants, hormone injections, some intrauterine devices, or vasectomised partner]); male patients agreeing to use condoms during the study and for 3 months after the end of the study/last dose of the investigational medicinal product (IMP), or patients having a partner who is using a highly efficient method of contraception as described above.

## Detailed exclusion criteria

Patients who met any of the following criteria will be excluded from study enrolment. Having malignant tumors other than colorectal adenocarcinomas (current or within the previous 5 years), with the exception for curatively treated non-melanoma skin cancer or in situ carcinoma of the cervix. Having received treatment for CRC within 14 days of the first study drug dosing in the main study. Evidence of central nervous system metastases. Uncontrolled bowel obstruction/inflammatory bowel disease. A history of cardiac disease, myocardial infarction or unstable angina in the preceding 6 months. Current severe chronic diarrhoea. Current chronic infection or uncontrolled serious illness causing immunodeficiency. Any current uncontrolled serious illness or medical condition. Any current major psychiatric disorder (e.g. major depression, psychosis). Participation in another clinical study with an investigational medical product [IMP] within 1 month prior to the start of the present study. A known intolerance to 5-FU therapy suggestive of dihydropyrimidine dehydrogenase deficiency. Suspicion of hypersensitivity or allergy to the IMP, or to products chemically related to the IMP (i.e. folate derivatives). Female patients who were pregnant or breast-feeding at study start. Previous treatment with arfolitixorin at any dose, regardless of indication treated and suspicion, as per Investigator’s judgement, of forthcoming need of any chemotherapeutic agents, antibodies or biologics other than those included in this protocol.

# Patient enrolment and treatment allocation

## **Table S1.** Patient enrollment and treatment allocation

| **Arm** | **Cohort^a^** | **Treatment description** | **Arfolitixorin dose (mg/m^2^)** | **Number**  **enrolled overall** | **Number included in PK analysis**  **(cycle 1/cycle 4)^a^** |
| --- | --- | --- | --- | --- | --- |
| 1 | 1 | 5-FU bolus (500 mg/m^2^)  + arfolitixorin | 30 | 4 | 4/3 |
|  | 2 |  | 60 | 3 | 2/2 |
|  | 8 |  | 120 | 3 | - |
|  | 9 |  | 240 | 3 | 2/2 |
| 2 | 4 | Oxaliplatin (85 mg/m^2^)  + 5-FU bolus (500 mg/m^2^)  + arfolitixorin | 30 | 4 | 1/1 |
|  | 5 |  | 60 | 6 | 4/2 |
| 3 | 6 | Irinotecan (180 mg/m^2^)  + 5-FU bolus (500 mg/m^2^)  + arfolitixorin | 30 | 5 | 4/3 |
|  | 7 |  | 60 | 3 | 2/2 |
| 4 | 12 | Oxaliplatin (85 mg/m^2^)  + 5-FU bolus (400 mg/m^2^)  and infusion (2,400 mg/m^2^)  + arfolitixorin | 60 (2 x 30 mg/m^2^) | 3 | 1/1 |
|  | 13 |  | 120 (2 x 60 mg/m^2^) | 8 | 3/2 |
|  | 14 |  | 240 (2 x 120 mg/m^2^) | 4 | 3/1 |
|  | 18 |  | 120 (2 x 60 mg/m^2^) | 22 | 7/7 |
| 5 | 15 | Oxaliplatin (85 mg/m^2^)  + bevacizumab (5 mg/kg)  + 5-FU bolus (400 mg/m^2^)  and infusion (2,400 mg/m^2^)  + arfolitixorin | 60 (2 x 30 mg/m^2^) | 5 | 2/3^b^ |
|  | 16 |  | 120 (2 x 60 mg/m^2^) | 11 | 6/6 |
| 6 | 19 | Irinotecan (180 mg/m^2^)  + 5-FU bolus (400 mg/m^2^) and infusion (2,400 mg/m^2^)  + arfolitixorin | 120 (2 x 60 mg/m^2^) | 21 | 10/8^†^ |
|  |  |  | Total | 105 | 51/43 |

^a^Cohorts 3, 10, 11 and 17 were originally specified in earlier versions of the study protocol.

^b^A patient was excluded from the PK calculations in cycle 1.

5-FU, 5-fluorouracil; PK, pharmacokinetics.

# Pharmacokinetic Results

Across arms 1−6, 53 patients contributed to the PK analysis, including 27 females and 26 males aged 32−84 years.

## [6R]-5,10-methylenetetrahydrofolate (active substance)

For the active substance [6R]-MTHF, all patients had a level below the LLOQ before administration of arfolitixorin in cycles 1 and 4. The t_max_ was reached at 10 minutes in arms 1−3, and at 40 minutes in arms 4−6 (Figure 4). [6R]-MTHF was eliminated rapidly, and the last detectable amount in blood was generally reached at ~1 hour post-administration. There was no evidence of [6R]-MTHF accumulating in plasma. Linear PK were generally observed for arms 1−3 (single bolus administration), in which the AUC_0−1h_ increased in proportion to the dose of arfolitixorin (30 to 240 mg/m^2^). There were no major differences between the levels of [6R]-MTHF in arm 1 (5-FU and arfolitixorin), arm 2 (oxaliplatin, 5-FU and arfolitixorin) or arm 3 (irinotecan, 5-FU and arfolitixorin). The C_10min_ was lower for treatment arms 4−6 than for arms 1−3, reflecting the lower concentration associated with two bolus injections (arms 4−6) instead of one. Lower dose-corrected mean serum concentrations of [6R]-MTHF were seen at C_10min_ in arm 5 (ARFOX and bevacizumab) compared with arm 4 (ARFOX) in patients given arfolitixorin 120 mg/m^2^, although the inter-individual variation was still relatively high (Table S2). No major differences in PK were observed between cycle 1 and cycle 4 at any of the dose levels of arfolitixorin.


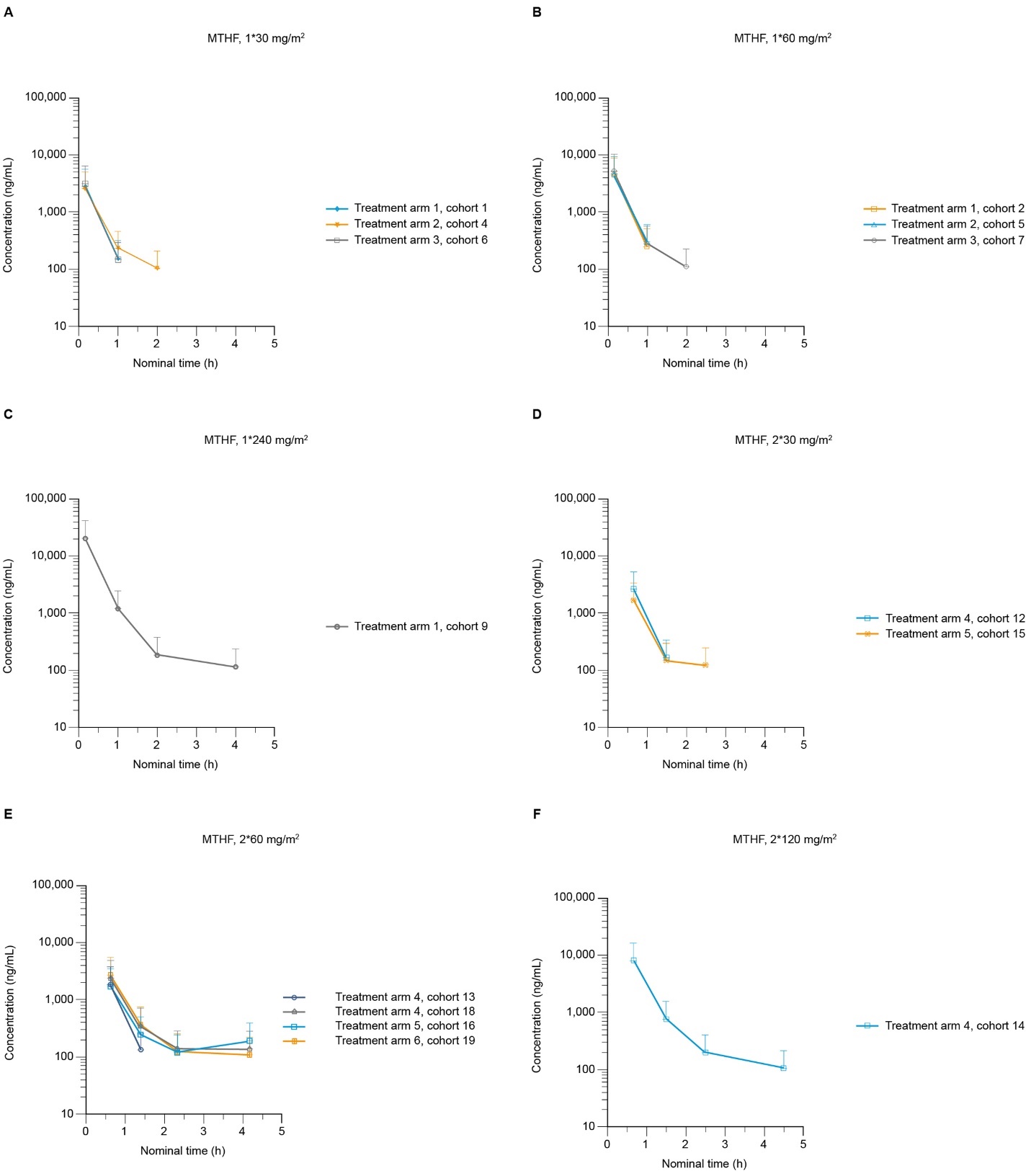


### **Figure S1.** Mean (± SD) plasma concentration versus time for arfolitixorin ([6R]-MTHF) (cycle 1 and cycle 4 combined data) divided by treatment arm according to arfolitixorin dose

(1*30 mg/m^2^ [A]; 1*60 mg/m^2^ [B]; 1*240 mg/m^2^ [C]; 2*30 mg/m^2^ [D]; 2*60 mg/m^2^ [E]; 2*120 mg/m^2^ [F]). For treatment arms 4−6, the doses of arfolitixorin were given as two bolus injections at 30 minutes apart, and the PK blood samples at 10 minutes, and 1, 2, and 4 hours were collected after the second administration of arfolitixorin, i.e. at 0, 40 minutes, and 1.5, 2.5, and 4.5 hours after first dose. In the above graphs, the nominal times are displayed as the same nominal times for treatment arms 1–3.

### **Table S2**. Statistics of main pharmacokinetic (PK) parameters of parent compound MTHF (arfolitixorin) after I.V. bolus administration (Cycle 1 and Cycle 4 combined)

|  | | | | |  | Analyte | | | |
| --- | --- | --- | --- | --- | --- | --- | --- | --- | --- |
|  |  |  |  |  |  | **MTHF** | | | |
| Arm | **Cohort** | | **Dose (mg/m^2^)** | |  | **C_10min_ (ng/mL)** | **AUC_0-1h_ (h*ng/mL)** | **C_10min_/Dose**  **((ng/mL)/mg)** | **AUC_0-1h_/Dose**  **((h*ng/mL)/mg)** |
| 1 | **1** | | **1*30** | | **N** | **7** | **7** | **7** | **7** |
|  | | | | | **Mean** | **2860** | **1460** | **47.6** | **24.2** |
|  | | | | | **SD** | **650** | **330** | **13** | **6.7** |
|  | | | | | **Median** | **3110** | **1560** | **47.3** | **23.7** |
| 1 | **2** | | **1*60** | | **N** | **4** | **3** | **4** | **3** |
|  | | | | | **Mean** | **4410** | **2370** | **40.1** | **21.6** |
|  | | | | | **SD** | **800** | **330** | **7.3** | **3.0** |
|  | | | | | **Median** | **4400** | **2560** | **40.0** | **23.2** |
| 1 | **9** | | **1*240** | | **N** | **4** | **4** | **4** | **4** |
|  | | | | | **Mean** | **21200** | **10600** | **47.3** | **23.7** |
|  | | | | | **SD** | **6400** | **3300** | **17** | **8.7** |
|  | | | | | **Median** | **22000** | **11000** | **48.9** | **24.5** |
| 2 | **4** | | **1*30** | | **N** | **2** | **2** | **2** | **2** |
|  | | | | | **Mean** | **2550** | **1440** | **40.5** | **22.8** |
|  | | | | | **SD** | **920** | **280** | **15** | **4.5** |
|  | | | | | **Median** | **2550** | **1440** | **40.5** | **22.8** |
| 2 | **5** | | **1*60** | | **N** | **6** | **5** | **6** | **5** |
|  | | | | | **Mean** | **4600** | **2710** | **43.8** | **25.7** |
|  | | | | | **SD** | **1100** | **310** | **9.7** | **2.9** |
|  | | | | | **Median** | **4710** | **2860** | **45.0** | **24.4** |
| 3 | **6** | | **1*30** | | **N** | **7** | **5** | **7** | **5** |
|  | | | | | **Mean** | **3170** | **1580** | **58.4** | **29.2** |
|  | | | | | **SD** | **440** | **250** | **7.4** | **3.4** |
|  | | | | | **Median** | **3290** | **1620** | **59.8** | **30.0** |
| 3 | **7** | | **1*60** | | **N** | **4** | **4** | **4** | **4** |
|  | | | | | **Mean** | **5120** | **2580** | **42.7** | **21.5** |
|  | | | | | **SD** | **1200** | **520** | **9.7** | **4.3** |
|  | | | | | **Median** | **5280** | **2710** | **44.0** | **22.6** |
| 4 | **12** | | **2*30** | | **N** | **2** | **NC** | **2** | **NC** |
|  | | | | | **Mean** | **2660** |  | **24.1** |  |
|  | | | | | **SD** | **320** |  | **2.9** |  |
|  | | | | | **Median** | **2660** |  | **24.1** |  |
| 4 | **13** | | **2*60** | | **N** | **5** | **NC** | **5** | **NC** |
|  | | | | | **Mean** | **2910** |  | **13.9** |  |
|  | | | | | **SD** | **1400** |  | **7.6** |  |
|  | | | | | **Median** | **2300** |  | **11.2** |  |
| 4 | **14** | | **2*120** | | **N** | **4** | **NC** | **4** | **NC** |
|  | | | | | **Mean** | **8070** |  | **19.7** |  |
|  | | | | | **SD** | **1900** |  | **4.9** |  |
|  | | | | | **Median** | **7910** |  | **19.8** |  |
| 4 | **18** | | **2*60** | | **N** | **14** | **NC** | **14** | **NC** |
|  | | | | | **Mean** | **3770** |  | **18.7** |  |
|  | | | | | **SD** | **1900** |  | **11** |  |
|  | | | | | **Median** | **3450** |  | **16.0** |  |
| 5 | **15** | | **2*30** | | **N** | **5** | **NC** | **5** | **NC** |
|  | | | | | **Mean** | **1890** |  | **16.8** |  |
|  | | | | | **SD** | **780** |  | **6.7** |  |
|  | | | | | **Median** | **1970** |  | **20.5** |  |
| 5 | **16** | | **2*60** | | **N** | **12** | **NC** | **12** | **NC** |
|  | | | | | **Mean** | **2590** |  | **12.1** |  |
|  | | | | | **SD** | **2200** |  | **10** |  |
|  | | | | | **Median** | **1900** |  | **8.92** |  |
| 6 | | **19** | | **2*60** | **N** | **18** | **NC** | **18** | **NC** |
|  | | | | | **Mean** | **4150** |  | **17.7** |  |
|  | | | | | **SD** | **2100** |  | **8.8** |  |
|  | | | | | **Median** | **4080** |  | **17.6** |  |
| N: Sum of patients in Cycle 1 and Cycle 4 | | | | | | | | | |
| MTHF, methyltetrahydrofolate; NC: Not calculated; SD, standard deviation. | | | | | | | | | |
| For treatment arms 4 - 6, C_10min_= 10 min after 2^nd^ dose, i.e. 40 min after 1^st^ dose | | | | | | | | | |

**Arm #1**: arfolitixorin (1*30, 1*60, 1*120 or 1*240 mg/m^2^) with 5-FU (500 mg/m^2^) alone

**Arm #2**: arfolitixorin (1*30 or 1*60 mg/m^2^) with oxaliplatin (85 mg/m^2^) and 5-FU (500 mg/m^2^)

**Arm #3**: arfolitixorin (1*30 or 1*60 mg/m^2^) with irinotecan (180 mg/m^2^) and 5-FU (500 mg/m^2^)

**Arm #4**: arfolitixorin (2*30, 2*60 or 2*120 mg/m^2^) with oxaliplatin (85 mg/m^2^) and 5-FU bolus + infusion (400 +

2400 mg/m^2^)

**Arm #5**: arfolitixorin (2*30 or 2*60 mg/m^2^) with oxaliplatin (85 mg/m^2^), 5-FU bolus + infusion (400 + 2400 mg/m^2^)

and bevacizumab (5 mg/kg).

**Arm #6**: arfolitixorin (2*60 mg/m^2^) with irinotecan (180 mg/m^2^), 5-FU bolus + infusion (400 + 2400 mg/m^2^).

## THF (metabolite)

For the metabolite THF, the t_max_ was reached 10 minutes after the first dose of arfolitixorin in arms 1−3, and 40 minutes after the first dose in arms 4−6. The formation and elimination of THF was rapid. The t_max_ was reached at 10 minutes post-administration in arms 1−3, and 40 minutes after the first dose (10 minutes after the second dose) in arms 4−6. The last detectable blood sample was generally at 2 hours post-administration for arfolitixorin 30 mg/m^2^, and low concentrations were evident at 4 hours for the other doses (Figure S2). PK parameters were consistent between chemotherapy cycles 1 and 4, and no major differences in the PK of THF were observed between any of the dose levels of arfolitixorin. In treatment arms 1−3, the AUC_0-1h_ and AUC_0-2h_ of THF increased in proportion to the dose increase of arfolitixorin from 30 to 60 mg/m^2^, demonstrating a linear dose-concentration relationship (Table S3). There was evidence of an exponential relationship between dose and concentration between 120 and 240 mg/m^2^ compared with lower doses, although PK for arfolitixorin was only evaluated in four patients on arfolitixorin 240 mg/m^2^ (Table S3). The plasma levels of THF were comparable 10 minutes after the single bolus in arms 1−3 and 10 minutes after the second bolus in arms 4−6.

| **A B** | |
| --- | --- |
| 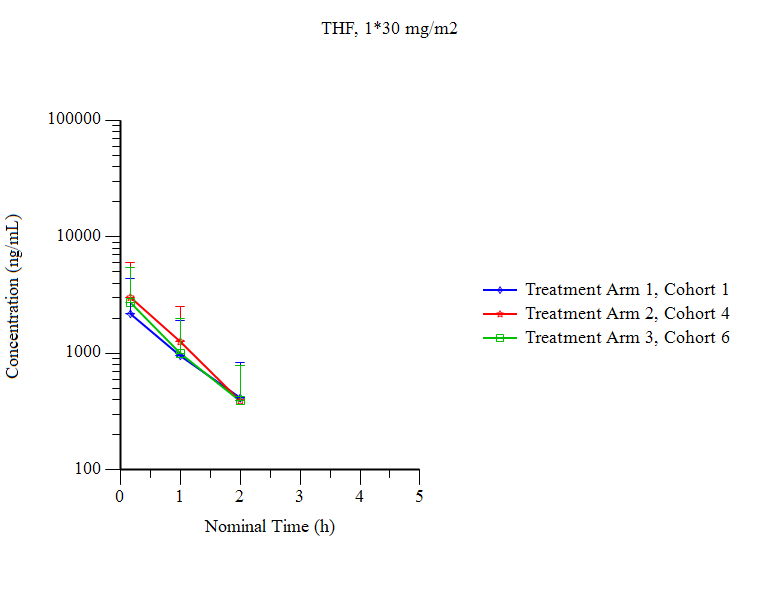 | 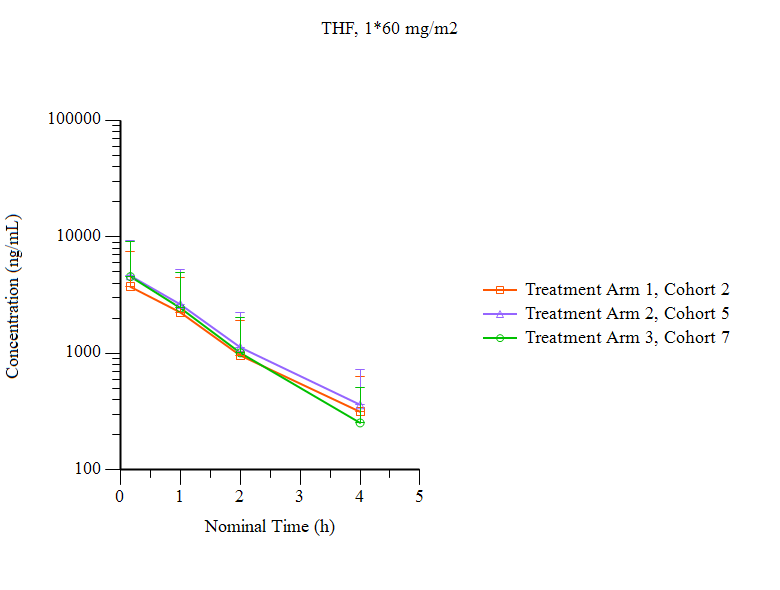 |
| **C** | **D** |
|  |  |
| 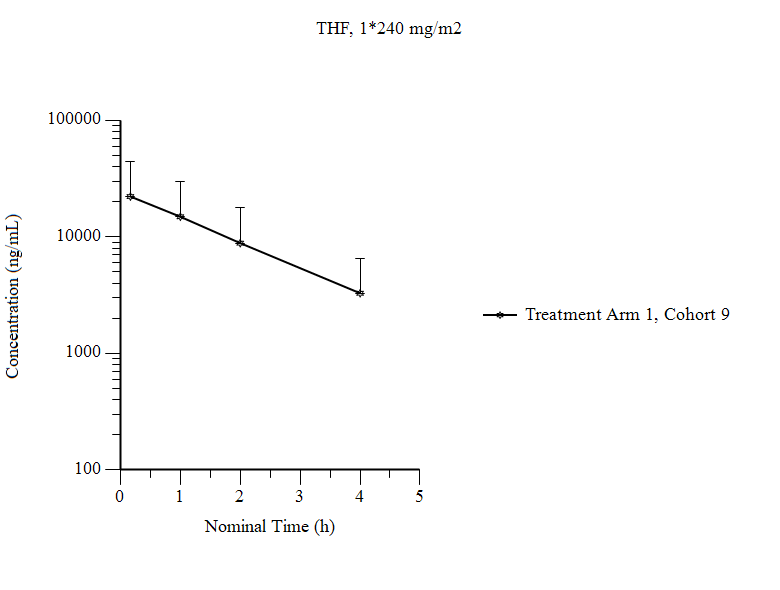 | 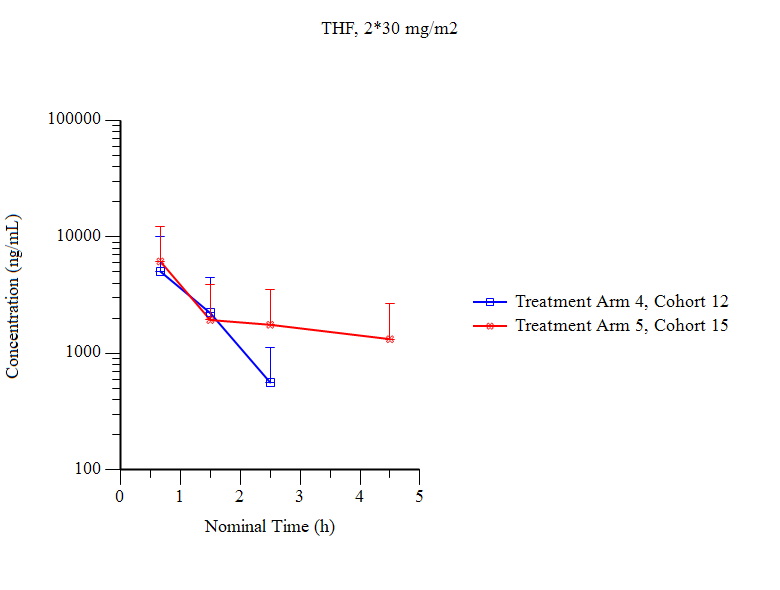 |
| **E**  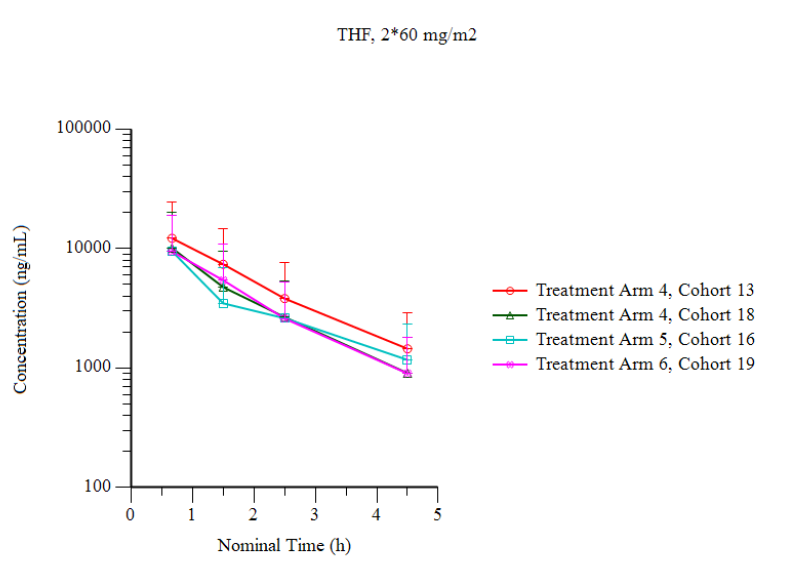 | **F**  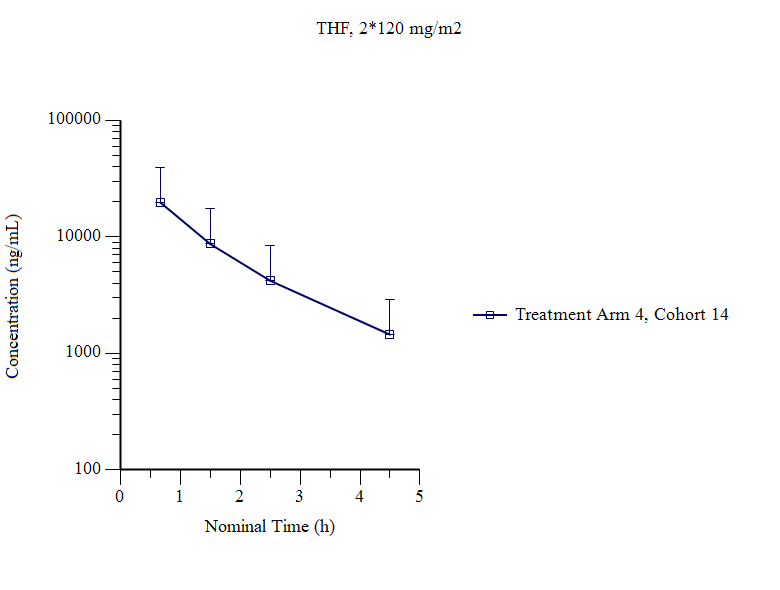 |

### **Figure S2**. Mean (± SD) plasma concentration vs time for tetrahydrofolate (THF) (Cycle 1 and Cycle 4 combined data) divided by treatment arm according to arfolitixorin dose

(1*30 mg/m^2^ [A]; 1*60 mg/m^2^ [B]; 1*240 mg/m^2^ [C]; 2*30 mg/m^2^ [D]; 2*60 mg/m^2^ [E]; 2*120 mg/m^2^ [F]. For treatment Arms 4, 5 and 6, the doses of arfolitixorin were given as two bolus injections at 0.5 hours apart, and the PK blood samples at 10 minutes, and 1, 2, and 4 hours were collected after the second administration of arfolitixorin, i.e. at 0, 40 minutes, and 1.5, 2.5 and 4.5 hours after first dose. In the above graphs, the nominal times are displayed as the same nominal times for treatment Arms 1–3.

Arm 1: arfolitixorin (1*30, 1*60, 1*120 or 1*240 mg/m^2^) with 5-FU (500 mg/m^2^) alone Arm 2: arfolitixorin (1*30 or 1*60 mg/m^2^) with oxaliplatin (85 mg/m^2^) and 5-FU (500 mg/m^2^). Arm 3: arfolitixorin (1*30 or 1*60 mg/m^2^) with irinotecan (180 mg/m^2^) and 5-FU (500 mg/m^2^). Arm 4: arfolitixorin (2*30, 2*60 or 2*120 mg/m^2^) with oxaliplatin (85 mg/m^2^) and 5-FU bolus + infusion (400 + 2,400 mg/m^2^). Arm 5: arfolitixorin (2*30 or 2*60 mg/m^2^) with oxaliplatin (85 mg/m^2^), 5-FU bolus + infusion (400 + 2,400 mg/m^2^) and bevacizumab (5 mg/kg). Arm 6: arfolitixorin (2*60 mg/m^2^) with irinotecan (180 mg/m^2^), 5-FU bolus + infusion (400 + 2400 mg/m^2^).

### **Table S3**. Statistics of main PK parameters of THF after IV bolus administration of arfolitixorin (Cycles 1 and 4 combined)

|  | | |  | **Analyte** | | | | | |
| --- | --- | --- | --- | --- | --- | --- | --- | --- | --- |
|  |  |  |  | **THF** | | | | | |
| **Arm** | **Cohort** | **Dose (mg/m^2^)** |  | **C_10min_ (ng/mL)** | **AUC_0-1h_ (h*ng/mL)** | **AUC_0-2h_**  **(h*ng/mL)** | **C_10min_/Dose**  **((ng/mL)/mg)** | **AUC_0-1h_/Dose**  **((h*ng/mL)/mg)** | **AUC_0-2h_/Dose**  **((h*ng/mL)/mg)** |
| **1** | **1** | **1*30** | **N** | **7** | **7** | **5** | **7** | **7** | **5** |
|  | | | **Mean** | **2200** | **1670** | **2550** | **36.6** | **27.8** | **44.2** |
|  | | | **SD** | **490** | **330** | **150** | **9.9** | **7.4** | **4.7** |
|  | | | **Median** | **2290** | **1710** | **2500** | **39.6** | **30.5** | **43.9** |
| **1** | **2** | **1*60** | **N** | **4** | **4** | **4** | **4** | **4** | **4** |
|  | | | **Mean** | **3740** | **3100** | **4600** | **34.0** | **28.2** | **41.8** |
|  | | | **SD** | **360** | **240** | **220** | **3.3** | **2.2** | **2.0** |
|  | | | **Median** | **3640** | **3010** | **4510** | **33.0** | **27.4** | **41.0** |
| **1** | **9** | **1*240** | **N** | **4** | **4** | **4** | **4** | **4** | **4** |
|  | | | **Mean** | **22700** | **19500** | **31600** | **50.5** | **43.6** | **70.5** |
|  | | | **SD** | **5700** | **5400** | **9500** | **16** | **14** | **25** |
|  | | | **Median** | **22200** | **19200** | **30900** | **49.1** | **42.7** | **68.8** |
| **2** | **4** | **1*30** | **N** | **2** | **2** | **2** | **2** | **2** | **2** |
|  | | | **Mean** | **3030** | **2290** | **3040** | **48.0** | **36.4** | **48.3** |
|  | | | **SD** | **110** | **75** | **110** | **1.7** | **1.2** | **1.8** |
|  | | | **Median** | **3030** | **2290** | **3040** | **48.0** | **36.4** | **48.3** |
| **2** | **5** | **1*60** | **N** | **6** | **6** | **6** | **6** | **6** | **6** |
|  | | | **Mean** | **4640** | **3860** | **5650** | **44.6** | **36.9** | **53.8** |
|  | | | **SD** | **530** | **650** | **1100** | **7.0** | **6.2** | **9.0** |
|  | | | **Median** | **4670** | **4160** | **6100** | **43.9** | **37.6** | **55.1** |
| **3** | **6** | **1*30** | **N** | **7** | **7** | **7** | **7** | **7** | **7** |
|  | | | **Mean** | **2720** | **1960** | **2610** | **49.8** | **35.9** | **47.8** |
|  | | | **SD** | **650** | **420** | **530** | **8.8** | **5.4** | **6.5** |
|  | | | **Median** | **2730** | **2030** | **2770** | **49.6** | **37.0** | **49.9** |
| **3** | **7** | **1*60** | **N** | **4** | **4** | **4** | **4** | **4** | **4** |
|  | | | **Mean** | **4520** | **3630** | **5280** | **37.6** | **30.2** | **44.0** |
|  | | | **SD** | **770** | **530** | **580** | **6.4** | **4.4** | **4.9** |
|  | | | **Median** | **4680** | **3740** | **5440** | **39.0** | **31.2** | **45.3** |
| **4** | **12** | **2*30** | **N** | **2** | **NC** | **NC** | **2** | **NC** | **NC** |
|  | | | **Mean** | **5030** |  |  | **45.7** |  |  |
|  | | | **SD** | **950** |  |  | **8.6** |  |  |
|  | | | **Median** | **5030** |  |  | **45.7** |  |  |
| **4** | **13** | **2*60** | **N** | **5** | **NC** | **NC** | **5** | **NC** | **NC** |
|  | | | **Mean** | **12500** |  |  | **59.0** |  |  |
|  | | | **SD** | **3200** |  |  | **20** |  |  |
|  | | | **Median** | **11400** |  |  | **55.3** |  |  |
| **4** | **14** | **2*120** | **N** | **4** | **NC** | **NC** | **4** | **NC** | **NC** |
|  | | | **Mean** | **19800** |  |  | **48.6** |  |  |
|  | | | **SD** | **4700** |  |  | **12** |  |  |
|  | | | **Median** | **21200** |  |  | **52.9** |  |  |
| **4** | **18** | **2*260** | **N** | **14** | **NC** | **NC** | **14** | **NC** | **NC** |
|  |  |  | **Mean** | **10100** |  |  | **49.6** |  |  |
|  |  |  | **SD** | **2200** |  |  | **13** |  |  |
|  |  |  | **Median** | **10300** |  |  | **50.6** |  |  |
| **5** | **15** | **2*30** | **N** | **5** | **NC** | **NC** | **5** | **NC** | **NC** |
|  | | | **Mean** | **5940** |  |  | **53.1** |  |  |
|  | | | **SD** | **1600** |  |  | **15** |  |  |
|  | | | **Median** | **6200** |  |  | **54.1** |  |  |
| **5** | **16** | **2*60** | **N** | **12** | **NC** | **NC** | **12** | **NC** | **NC** |
|  | | | **Mean** | **9610** |  |  | **45.2** |  |  |
|  | | | **SD** | **5100** |  |  | **24** |  |  |
|  | | | **Median** | **9610** |  |  | **45.3** |  |  |
| **6** | **19** | **2*60** | **N** | **18** | **NC** | **NC** | **18** | **NC** | **NC** |
|  | | | **Mean** | **9290** |  |  | **40.0** |  |  |
|  | | | **SD** | **2800** |  |  | **13** |  |  |
|  | | | **Median** | **9350** |  |  | **37.1** |  |  |
| N: Sum of patients in Cycle 1 and Cycle 4. | | | | | | | | | |
| NC: Not calculated; SD, standard deviation; THF, tetrahydrofolate. | | | | | | | | | |
| For treatment arms 4 - 6, C_10min_= 10 min after 2nd dose, i.e. 40 min after 1st dose | | | | | | | | | |

**Arm #1**: arfolitixorin (1*30, 1*60, 1*120 or 1*240 mg/m^2^) with 5-FU (500 mg/m^2^) alone

**Arm #2**: arfolitixorin (1*30 or 1*60 mg/m^2^) with oxaliplatin (85 mg/m^2^) and 5-FU (500 mg/m^2^)

**Arm #3**: arfolitixorin (1*30 or 1*60 mg/m^2^) with irinotecan (180 mg/m^2^) and 5-FU (500 mg/m^2^)

**Arm #4**: arfolitixorin (2*30, 2*60 or 2*120 mg/m^2^) with oxaliplatin (85 mg/m^2^) and 5-FU bolus + infusion (400 +

2400 mg/m^2^)

**Arm #5**: arfolitixorin (2*30 or 2*60 mg/m^2^) with oxaliplatin (85 mg/m^2^), 5-FU bolus + infusion (400 + 2400 mg/m^2^)

and bevacizumab (5 mg/kg).

**Arm #6**: arfolitixorin (2*60 mg/m^2^) with irinotecan (180 mg/m^2^), 5-FU bolus + infusion (400 + 2400 mg/m^2^).

## Methyl-THF (metabolite)

The t_max_ of the metabolite methyl-THF was generally reached 1−2 hours after administration of arfolitixorin but there was some variability up to a maximum of 4 hours post-dose in some individuals, indicating a relatively slow rate of formation and elimination (Figure S3). PK parameters for methyl-THF were consistent between cycles, and there were no major differences in parameters between cycles 1 and 4 for any of the doses of arfolitixorin. The AUC_0−4h_ for methyl-THF increased linearly between arfolitixorin doses of 30 and 250 mg/m^2^ (Table S4). Some variation was evident in the maximum concentration of methyl-THF in arms 4−6, but levels were generally comparable to the maximum concentrations observed following a single bolus dose of arfolitixorin in arms 1−3.

**A B**

|  | |
| --- | --- |
| 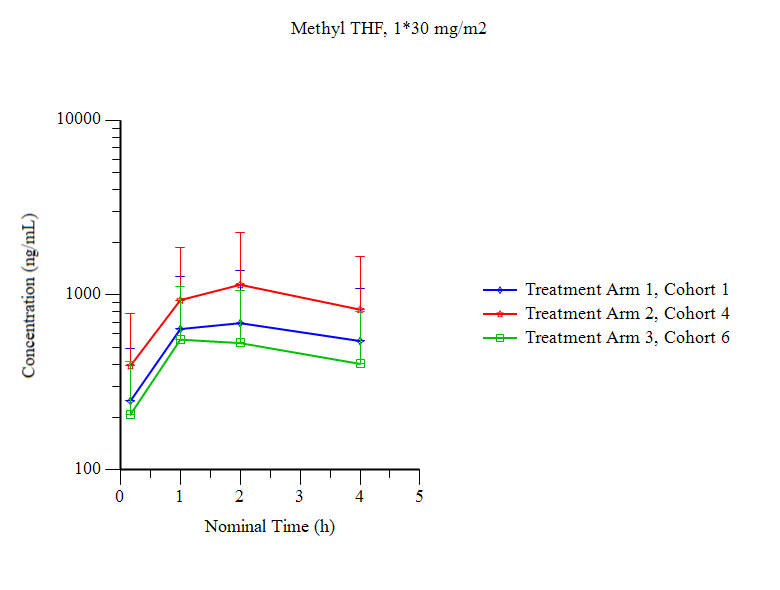 | 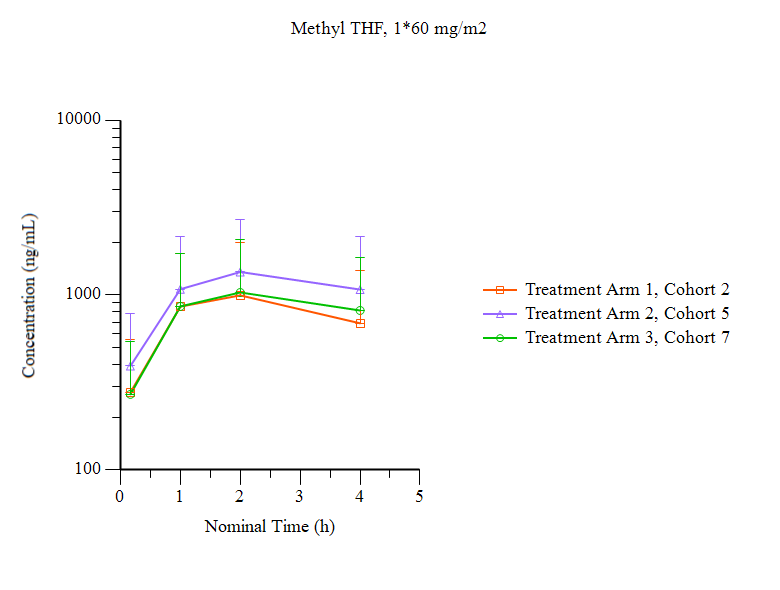 |
| **C** | **D** |
| 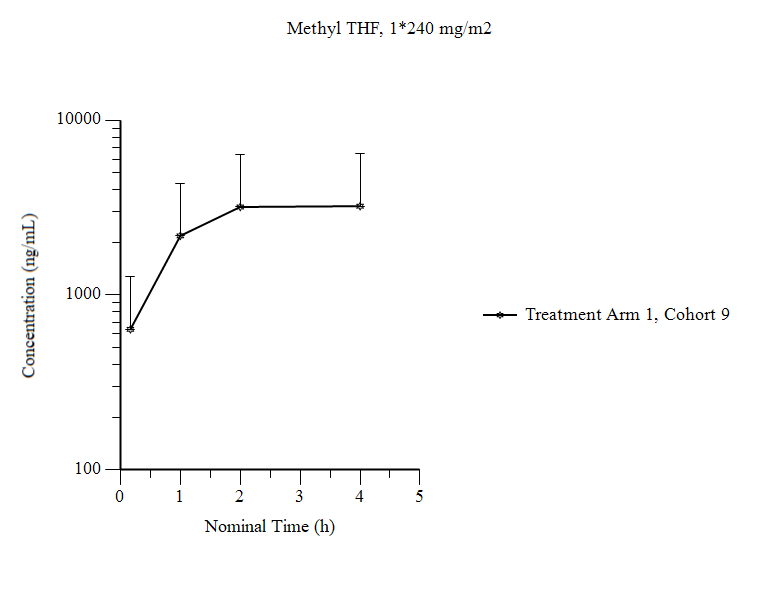 | 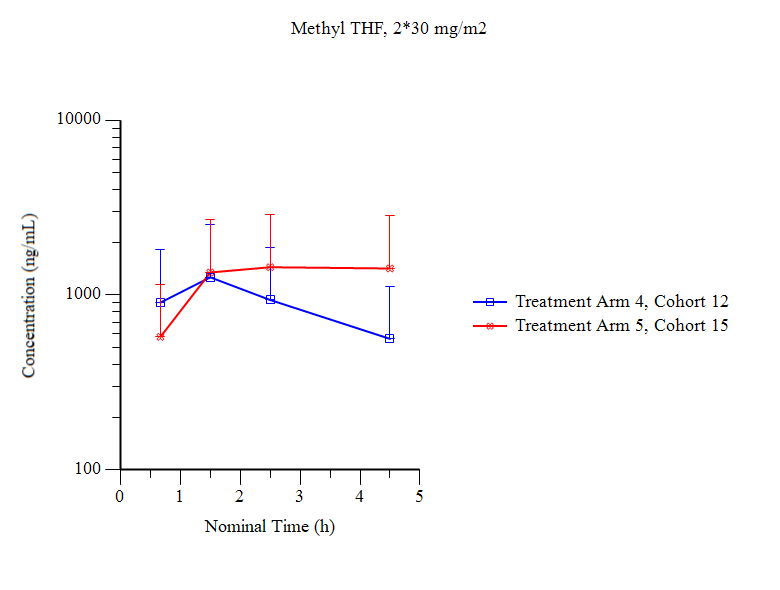 |
| **E** | **F** |
| 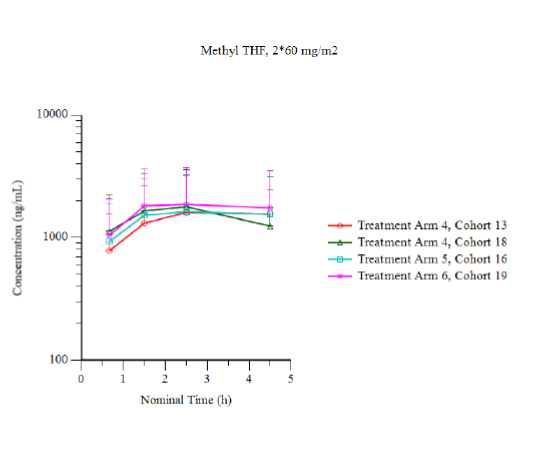 | 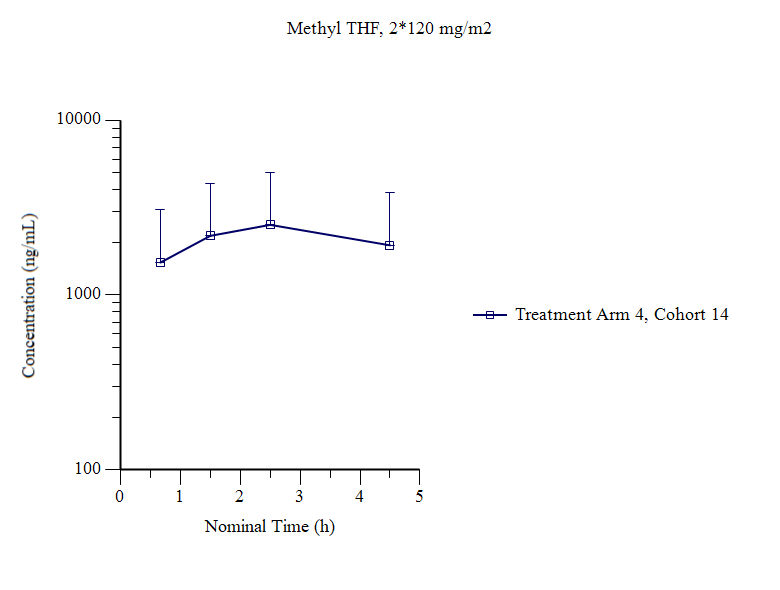 |

### **Figure S3**. Mean (± SD) plasma concentration vs time for methyltetrahydrofolate (Methyl THF) (Cycle 1 and Cycle 4 combined data) divided by treatment arm according to arfolitixorin dose

(1*30 mg/m^2^ [A]; 1*60 mg/m^2^ [B]; 1*240 mg/m^2^ [C]; 2*30 mg/m^2^ [D]; 2*60 mg/m^2^ [E]; 2*120 mg/m^2^ [F]). For treatment Arms 4, 5 and 6, the doses of arfolitixorin were given as two bolus injections at 0.5 hours apart, and the PK blood samples at 10 minutes, and 1, 2, and 4 hours were collected after the second administration of arfolitixorin, i.e. at 0, 40 minutes, and 1.5, 2.5 and 4.5 hours after first dose. In the graphs for Figure S2 graphs, the nominal times are displayed as the same nominal times for treatment Arms 1–3.

Arm 1: arfolitixorin (1*30, 1*60, 1*120 or 1*240 mg/m^2^) with 5-FU (500 mg/m^2^) alone Arm 2: arfolitixorin (1*30 or 1*60 mg/m^2^) with oxaliplatin (85 mg/m^2^) and 5-FU (500 mg/m^2^). Arm 3: arfolitixorin (1*30 or 1*60 mg/m^2^) with irinotecan (180 mg/m^2^) and 5-FU (500 mg/m^2^). Arm 4: arfolitixorin (2*30, 2*60 or 2*120 mg/m^2^) with oxaliplatin (85 mg/m^2^) and 5-FU bolus + infusion (400 + 2,400 mg/m^2^). Arm 5: arfolitixorin (2*30 or 2*60 mg/m^2^) with oxaliplatin (85 mg/m^2^), 5-FU bolus + infusion (400 + 2,400 mg/m^2^) and bevacizumab (5 mg/kg). Arm 6: arfolitixorin (2*60 mg/m^2^) with irinotecan (180 mg/m^2^), 5-FU bolus + infusion (400 + 2400 mg/m^2^).

### **Table S4**. Statistics of main PK parameters of Methyl-THF after IV bolus administration of arfolitixorin (Cycle 1 and Cycle 4 combined).

|  | | | | |  | **Analyte** | | | | |
| --- | --- | --- | --- | --- | --- | --- | --- | --- | --- | --- |
|  |  |  |  |  |  | **Methyl THF** | | | | |
| **Arm** | **Cohort** | | **Dose (mg/m^2^)** | |  | **T_max_(h)** | **C_max_(ng/mL)** | **AUC_0-4h_**  **(h*ug/L)** | **C_max_/Dose**  **(ng/mL/mg)** | **AUC_0-4h_/Dose**  **((h*ng/mL)/mg)** |
| **1** | **1** | | **1*30** | | **N** | **7** | **7** | **7** | **7** | **7** |
|  | | | | | **Mean** | **1.74** | **679** | **2250** | **11.1** | **36.7** |
|  | | | | | **SD** | **0.49** | **110** | **360** | **1.3** | **3.9** |
|  | | | | | **Median** | **2.02** | **643** | **2250** | **11.2** | **38.0** |
| **1** | **2** | | **1*60** | | **N** | **4** | **4** | **4** | **4** | **4** |
|  | | | | | **Mean** | **1.74** | **1020** | **3130** | **9.27** | **28.4** |
|  | | | | | **SD** | **0.49** | **260** | **690** | **2.4** | **6.3** |
|  | | | | | **Median** | **1.98** | **1100** | **3330** | **10.0** | **30.3** |
| **1** | **9** | | **1*240** | | **N** | **4** | **4** | **4** | **4** | **4** |
|  | | | | | **Mean** | **3.04** | **3640** | **10500** | **8.06** | **23.1** |
|  | | | | | **SD** | **1.1** | **490** | **760** | **1.5** | **2.4** |
|  | | | | | **Median** | **3.06** | **3420** | **10400** | **7.54** | **22.1** |
| **2** | **4** | | **1*30** | | **N** | **2** | **2** | **2** | **2** | **2** |
|  | | | | | **Mean** | **1.51** | **1210** | **3610** | **19.1** | **57.3** |
|  | | | | | **SD** | **0.72** | **280** | **610** | **4.4** | **9.8** |
|  | | | | | **Median** | **1.51** | **1210** | **3610** | **19.1** | **57.3** |
| **2** | **5** | | **1*60** | | **N** | **6** | **6** | **6** | **6** | **6** |
|  | | | | | **Mean** | **1.90** | **1370** | **4290** | **13.2** | **41.3** |
|  | | | | | **SD** | **0.41** | **79** | **340** | **1.6** | **5.2** |
|  | | | | | **Median** | **2.00** | **1350** | **4340** | **13.3** | **41.6** |
| **3** | **6** | | **1*30** | | **N** | **7** | **7** | **7** | **7** | **7** |
|  | | | | | **Mean** | **1.45** | **606** | **1800** | **11.2** | **33.1** |
|  | | | | | **SD** | **0.53** | **50** | **230** | **0.41** | **2.8** |
|  | | | | | **Median** | **1.12** | **610** | **1740** | **11.3** | **33.7** |
| **3** | **7** | | **1*60** | | **N** | **4** | **4** | **4** | **4** | **4** |
|  | | | | | **Mean** | **2.02** | **1040** | **3330** | **8.66** | **27.7** |
|  | | | | | **SD** | **0.033** | **120** | **230** | **1.0** | **1.9** |
|  | | | | | **Median** | **2.00** | **1080** | **3390** | **9.00** | **28.3** |
| **4** | **12** | | **2*30** | | **N** | **2** | **2** | **NC** | **2** | **NC** |
|  | | | | | **Mean** | **1.59** | **1260** |  | **11.5** |  |
|  | | | | | **SD** | **0.059** | **280** |  | **2.6** |  |
|  | | | | | **Median** | **1.59** | **1260** |  | **11.5** |  |
| **4** | **13** | | **2*60** | | **N** | **5** | **5** | **NC** | **5** | **NC** |
|  | | | | | **Mean** | **3.01** | **1540** |  | **7.16** |  |
|  | | | | | **SD** | **1.6** | **470** |  | **2.3** |  |
|  | | | | | **Median** | **2.62** | **1520** |  | **6.39** |  |
| **4** | **14** | | **2*120** | | **N** | **4** | **4** | **NC** | **4** | **NC** |
|  | | | | | **Mean** | **3.07** | **2540** |  | **6.21** |  |
|  | | | | | **SD** | **0.98** | **250** |  | **0.74** |  |
|  | | | | | **Median** | **2.61** | **2480** |  | **6.05** |  |
| **4** | | **18** | | **2*60** | **N** | **14** | **14** | **NC** | **14** | **NC** |
|  | | | | | **Mean** | **2.18** | **1920** |  | **9.47** |  |
|  | | | | | **SD** | **0.83** | **520** |  | **3.0** |  |
|  | | | | | **Median** | **2.23** | **1840** |  | **9.31** |  |
| **5** | **15** | | **2*30** | | **N** | **5** | **5** | **NC** | **5** | **NC** |
|  | | | | | **Mean** | **3.34** | **1450** |  | **13.0** |  |
|  | | | | | **SD** | **1.5** | **440** |  | **4.2** |  |
|  | | | | | **Median** | **3.98** | **1430** |  | **14.9** |  |
| **5** | **16** | | **2*60** | | **N** | **12** | **12** | **NC** | **12** | **NC** |
|  | | | | | **Mean** | **3.39** | **1750** |  | **8.24** |  |
|  | | | | | **SD** | **1.3** | **430** |  | **2.3** |  |
|  | | | | | **Median** | **3.56** | **1720** |  | **7.81** |  |
| **6** | | **19** | | **2*60** | **N** | **18** | **18** | **NC** | **18** | **NC** |
|  | | | | | **Mean** | **2.65** | **2040** |  | **8.86** |  |
|  | | | | | **SD** | **1.2** | **480** |  | **2.5** |  |
|  | | | | | **Median** | **2.47** | **1910** |  | **8.40** |  |
| N: Sum of patients in Cycle 1 and Cycle 4. | | | | | | | | | | |
| Methyl THF, Methyl-tetrahydrofolate; NC: Not calculated; SD, standard deviation. | | | | | | | | | | |

**Arm #1**: arfolitixorin (1*30, 1*60, 1*120 or 1*240 mg/m^2^) with 5-FU (500 mg/m^2^) alone

**Arm #2**: arfolitixorin (1*30 or 1*60 mg/m^2^) with oxaliplatin (85 mg/m^2^) and 5-FU (500 mg/m^2^)

**Arm #3**: arfolitixorin (1*30 or 1*60 mg/m^2^) with irinotecan (180 mg/m^2^) and 5-FU (500 mg/m^2^)

**Arm #4**: arfolitixorin (2*30, 2*60 or 2*120 mg/m^2^) with oxaliplatin (85 mg/m^2^) and 5-FU bolus + infusion (400 +

2400 mg/m^2^)

**Arm #5**: arfolitixorin (2*30 or 2*60 mg/m^2^) with oxaliplatin (85 mg/m^2^), 5-FU bolus + infusion (400 + 2400 mg/m^2^)

and bevacizumab (5 mg/kg).

**Arm #6**: arfolitixorin (2*60 mg/m^2^) with irinotecan (180 mg/m^2^), 5-FU bolus + infusion (400 + 2400 mg/m^2^).
